# Synthesis, Characterization, DFT Studies of Novel Cu(II), Zn(II), VO(II), Cr(III), and La(III) Chloro-Substituted Schiff Base Complexes: Aspects of Its Antimicrobial, Antioxidant, Anti-Inflammatory, and Photodegradation of Methylene Blue

**DOI:** 10.3390/molecules28124777

**Published:** 2023-06-15

**Authors:** Laila H. Abdel-Rahman, Maram T. Basha, Badriah Saad Al-Farhan, Walaa Alharbi, Mohamed R. Shehata, Noura O. Al Zamil, Doaa Abou El-ezz

**Affiliations:** 1 Chemistry Department, Faculty of Science, Sohag University, Sohag 82534, Egypt; 2 Department of Chemistry, College of Science, University of Jeddah, Jeddah 21589, Saudi Arabia; 3 Chemistry Department, Faculty of Girls for Science, King Khalid University, Abha 61421, Saudi Arabia; 4 Department of Chemistry, Science and Arts College, King Abdulaziz University, Rabigh 21911, Saudi Arabia; wnhalharbe@kau.edu.sa; 5 Chemistry Department, Faculty of Science, Cairo University, Giza 12613, Egypt; mrshehata@sci.cu.edu.eg; 6 Department of Chemistry, College of Science, Imam Abdurrahman Bin Faisal University, P.O. Box 1982, Dammam 31441, Saudi Arabia; nalzamil@iau.edu.sa; 7 Department of Pharmacology and Toxicology, Faculty of Pharmacy, October University for Modern Sciences and Arts (MSA University), Giza 12566, Egypt

**Keywords:** Schiff bases, DFT, antimicrobial, antioxidant, anti-inflammatory, DNA-binding, photodegradation

## Abstract

A new chlorobenzylidene imine ligand, (E)-1-((5-chloro-2-hydroxybenzylidene)amino) naphthalen-2-ol (HL), and its [Zn(L)(NO_3_)(H_2_O)_3_], [La(L)(NO_3_)_2_(H_2_O)_2_], [VO(L)(OC_2_H_5_)(H_2_O)_2_], [Cu(L)(NO_3_)(H_2_O)_3_], and [Cr(L)(NO_3_)_2_(H_2_O)_2_], complexes were synthesized and characterized. The characterization involved elemental analysis, FT-IR, UV/Vis, NMR, mass spectra, molar conductance, and magnetic susceptibility measurements. The obtained data confirmed the octahedral geometrical structures of all metal complexes, while the [VO(L)(OC_2_H_5_)(H_2_O)_2_] complex exhibited a distorted square pyramidal structure. The complexes were found to be thermally stable based on their kinetic parameters determined using the Coats–Redfern method. The DFT/B3LYP technique was employed to calculate the optimized structures, energy gaps, and other important theoretical descriptors of the complexes. In vitro antibacterial assays were conducted to evaluate the complexes’ potential against pathogenic bacteria and fungi, comparing them to the free ligand. The compounds exhibited excellent fungicidal activity against *Candida albicans* ATCC: 10231 (*C. albicans*) and *Aspergillus negar* ATCC: 16404 (*A. negar*), with inhibition zones of HL, [Zn(L)(NO_3_)(H_2_O)_3_], and [La(L)(NO_3_)_2_(H_2_O)_2_] three times higher than that of the Nystatin antibiotic. The DNA binding affinity of the metal complexes and their ligand was investigated using UV-visible, viscosity, and gel electrophoresis methods, suggesting an intercalative binding mode. The absorption studies yielded K_b_ values ranging from 4.40 × 10^5^ to 7.30 × 10^5^ M^−1^, indicating high binding strength to DNA comparable to ethidium bromide (value 10^7^ M^−1^). Additionally, the antioxidant activity of all complexes was measured and compared to vitamin C. The anti-inflammatory efficacy of the ligand and its metal complexes was evaluated, revealing that [Cu(L)(NO_3_)(H_2_O)_3_] exhibited the most effective activity compared to ibuprofen. Molecular docking studies were conducted to explore the binding nature and affinity of the synthesized compounds with the receptor of *Candida albicans* oxidoreductase/oxidoreductase INHIBITOR (PDB ID: 5V5Z). Overall, the combined findings of this work demonstrate the potential of these new compounds as efficient fungicidal and anti-inflammatory agents. Furthermore, the photocatalytic effect of the Cu(II) Schiff base complex/GO was examined.

## 1. Introduction

Schiff bases are significant ligands in coordination chemistry due to their ease of synthesis and the ability to modify them both sterically and electronically [1]. Coordination chemistry relies on the multicentricity of ligands, making ligands with mixed-donor atoms potentially useful in coordination complexes in various fields such as photochemistry, catalysis, and biological systems [2]. The hard and soft binding properties of nitrogen and sulfur donor atoms, respectively, have attracted increasing interest in coordination chemistry [3].

Copper and zinc, being essential nutrients for biological systems, have crucial roles in various physiological processes [3]. Copper contributes to the development of connective tissues, bones, and neurons, as well as hemoglobin synthesis [4]. Salicylaldehyde derivatives containing halo-atoms in the aromatic ring have shown promising biological activities, including antibacterial and antifungal properties. Based on these findings, Schiff bases of 5-chloro-salicylaldehyde were synthesized and evaluated for their antimicrobial properties [5]. Previous studies have demonstrated the notable in vitro antibacterial, anticancer, and antioxidant effects of copper compounds [6]. Zinc is known to be involved in insulin as a cofactor and plays a role in insulin action control. It is also an essential component of more than 200 functional proteins. Zinc complexes have been investigated for their potential anti-inflammatory, anti-proliferative, antibacterial, anti-diabetic, and radical scavenging effects [7].

Selected chromium complexes have been examined for their anti-inflammatory, antiproliferative, antibacterial, antifungal, and antioxidant activities. Chromium, especially chromium(III), has shown certain biological activities in regulating carbohydrates and lipid metabolism [8]. A low-chromium diet can lead to adverse effects such as glucose intolerance, growth disorders, and diminished longevity. Oxovanadium complexes have attracted researchers’ attention due to their well-known biological actions [9,10]. Vanadium complexes with different oxidation states have been successfully synthesized using various ligands, and their magnetic characteristics, anti-diabetic, anti-inflammatory, and catalytic activities have been evaluated. However, there are limited reports on the antibacterial activity of vanadium-based compounds [11,12,13]. The lanthanum element and its complexes have gained significant attention in biological research due to their potential applications in various aspects of biomedical activity [14]. Lanthanum ions can interact with proteins and enzymes, influencing their structure, function, and activity. Furthermore, lanthanum complexes can provide valuable insights into the mechanisms of biological processes and aid in drug discovery [15]. Lanthanum complexes have shown promising antimicrobial properties against various microorganisms, including bacteria and fungi. These complexes can inhibit microbial growth and offer potential alternatives to conventional antimicrobial agents [16,17].

The objectives of this study include the synthesis of a novel Schiff base ligand, specifically the chlorobenzylidene imine ligand, and its corresponding complexes. Furthermore, the aim is to characterize these compounds using various physical and spectral analytical techniques, as well as investigate their biological activity. To validate the experimental findings, the DFT/B3LYP method was employed. Moreover, the study examines the photocatalytic effect of the Cu(II) Schiff base complex/GO.

## 2. Results and Discussion

The HL ligand and its metal complexes, including [Cu(L)(NO_3_)(H_2_O)_3_], [Zn(L)(NO_3_)(H_2_O)_3_], [VO(L)(OC_2_H_5_)(H_2_O)_2_], [Cr(L)(NO_3_)_2_(H_2_O)_2_], and [La(L)(NO_3_)_2_(H_2_O)_2_], exhibit excellent stability at room temperature and are non-hygroscopic. HL itself is soluble in various solvents such as methanol, ethanol, DMF, DMSO, THF, and chloroform. On the other hand, the [Cu(L)(NO_3_)(H_2_O)_3_], [Zn(L)(NO_3_)(H_2_O)_3_], [VO(L)(OC_2_H_5_)(H_2_O)_2_], [Cr(L)(NO_3_)_2_(H_2_O)_2_], and [La(L)(NO_3_)_2_(H_2_O)_2_] complexes demonstrate solubility in DMF and DMSO, while being sparingly soluble in methanol and ethanol. Table 1 presents the elemental analysis data for the synthesized compounds, which show a good agreement with the calculated values. The molar conductance values of the metal complexes (Λ_M_ = 9.62–19.3 Ω^−1^ cm^2^ mol^−1^) indicate low conductivity, suggesting the absence of counter ions outside the coordination sphere. Moreover, the complexes are determined to be non-electrolytes, as shown in Table 1.

### 2.1. ^1^HNMR Spectra

**Figure 1:**
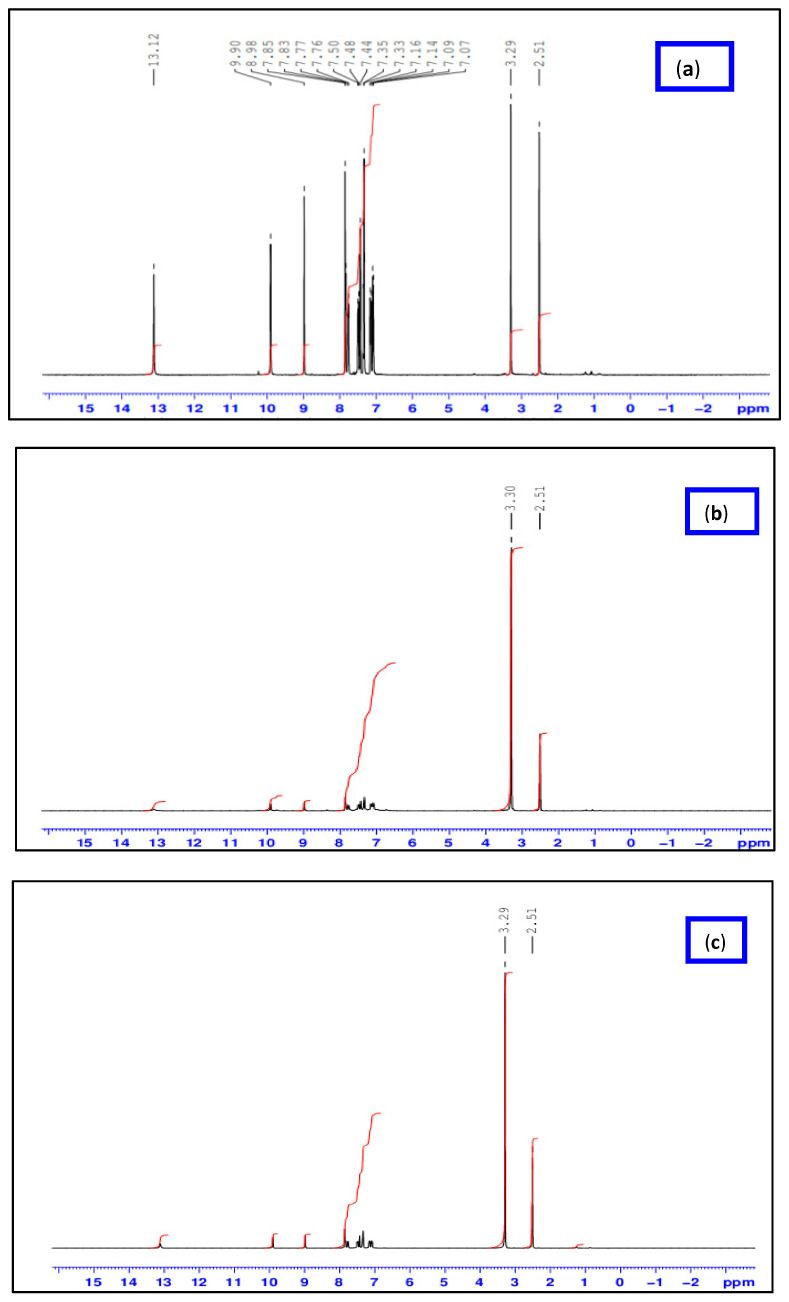
^1^HNMR spectra of HL ligand (**a**) and its [Zn(L)(NO_3_)(H_2_O)_3_] (**b**) and [La(L)(NO_3_)_2_(H_2_O)_2_] (**c**) complexes.

Figure 1 illustrates a singlet signal observed at 9.90 ppm in the ^1^H NMR spectrum of the HL ligand, corresponding to the CH=N proton. The aromatic protons of the phenyl and naphthalene rings, consisting of nine protons, display signals in the range of 7.0–8.98 ppm, indicating their presence. Furthermore, the phenolic protons are represented by a singlet signal observed at 13.12 ppm in the ligand’s ^1^H-NMR spectrum [18]. Upon examining the ^1^H NMR spectra of the diamagnetic [Zn(L)(NO_3_)(H_2_O)_3_] and [La(L)(NO_3_)_2_(H_2_O)_2_] complexes, it is evident that the aromatic protons appear as multiples compared to the parent ligand. Additionally, the azomethine (CH=N) proton experiences a downfield shift, appearing at 9.23 and 8.98 ppm for [Zn(L)(NO_3_)(H_2_O)_3_] and [La(L)(NO_3_)_2_(H_2_O)_2_], respectively. These observations suggest that the coordination of Zn(II) and La(III) ions with the ligand induces changes in the chemical environment of the aromatic and azomethine protons. Moreover, the absence of the OH proton signal, which was initially observed at 13.12 ppm in the ^1^H NMR spectra of the HL ligand, indicates its involvement in the coordination process with Zn(II) and La(III) ions. These findings provide evidence that the HL Schiff base coordinates with Zn(II) and La(III) ions through the azomethine nitrogen and phenolic oxygen atoms.

### 2.2. FT-IR

Figure S1 and Table 2 present the FT-IR spectra of the HL ligand and its complexes. In our analysis, a strong and sharp band at 1658 cm^−1^, corresponding to the stretching vibration of the azomethine group, confirms the synthesis of the Schiff base ligand [19]. As observed, the stretching frequency of the azomethine group shifts to lower frequencies (1597 and 1622 cm^−1^), indicating the coordination of the nitrogen atom of the azomethine with the metal atom in the complexes. This observation aligns with a study conducted by Abd El-Halim et al., where a similar red shift of the azomethine band (ν_(CH=N)_) by 11–27 cm^−1^ was observed in the complex spectra, indicating its involvement in coordination. Additionally, the formation of the C-O-M bond results in a redshift of ν_C-O_ by 4–31 cm^−1^, confirming the participation of the phenolic oxygen in complexation [20].

Notably, new peaks appear in the complexes between 570 and 659 cm^−1^ and 432–459 cm^−1^, indicating the presence of ν_M-O_ and ν_M-N_ vibrations. These results provide clear evidence of the Schiff base’s bidentate coordination mechanism, utilizing the N-azomethine and phenolic oxygen for metal coordination. The broad band observed between 3444 and 3327 cm^−1^ confirms the presence of coordinated water molecules in the complexes. Moreover, new bands appearing at 943–974 cm^−1^ in all metal complexes further confirm the existence of coordinated water. The band observed in the region of 1364 to 1381 cm^−1^ may correspond to the monovalent nitrate group coordinated with the metal ions, consistent with the findings of the complexes’ electrical conductivity. The bands between 615 and 697 cm^−1^ can be attributed to the ν_(C-Cl)_ vibration [18,21].

### 2.3. Mass Spectra

**Scheme 1:**
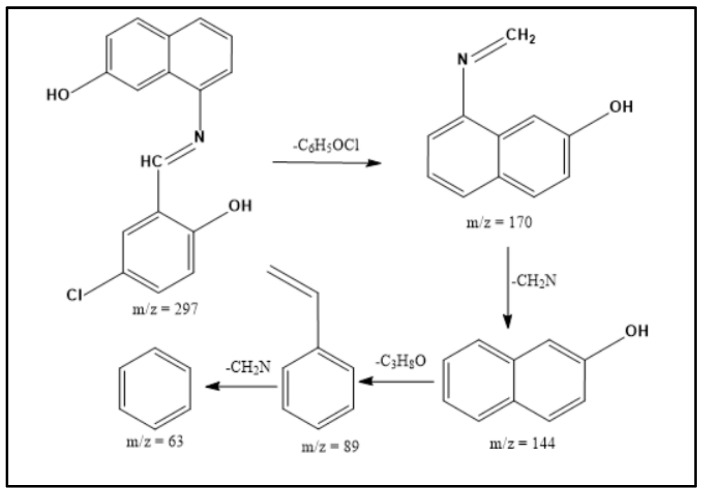
Mass fragmentation pattern of the HL Schiff base ligand.

**Scheme 2:**
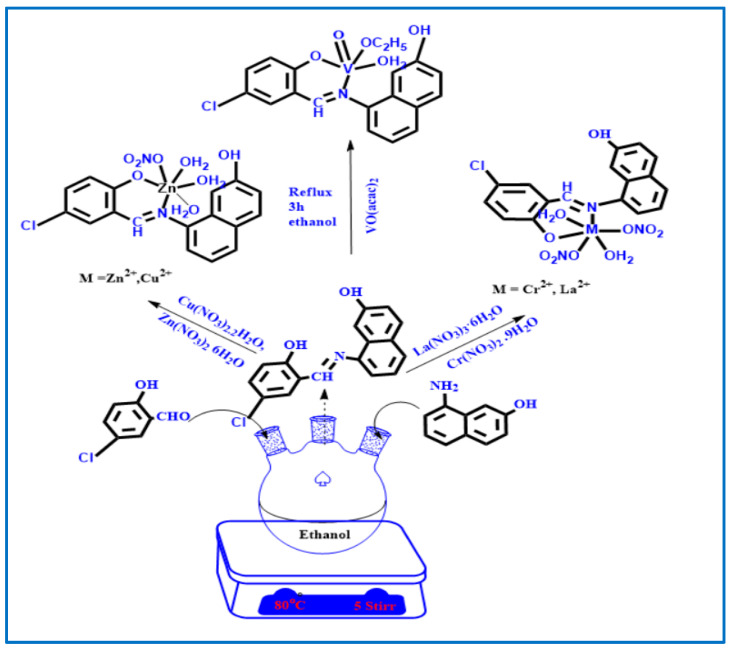
Schematic representation for the synthesis of HL Schiff base ligand and its metal complexes.

**Figure 2:**
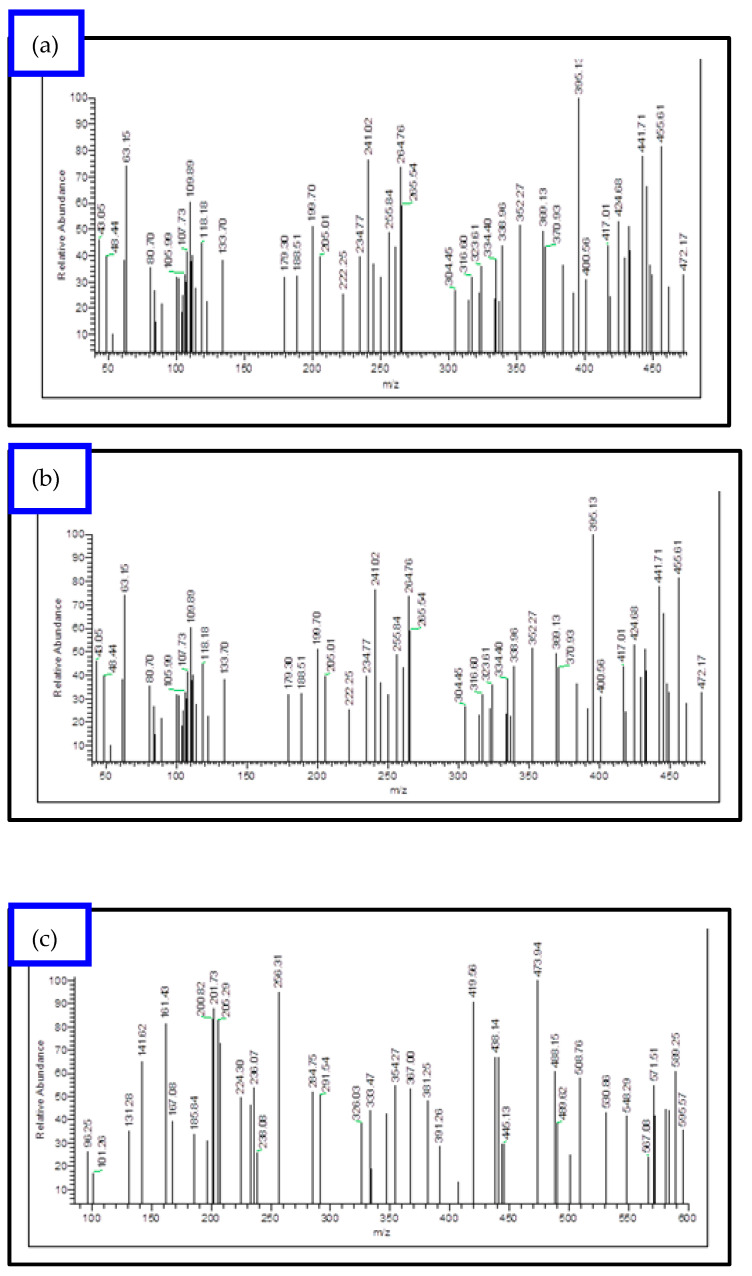

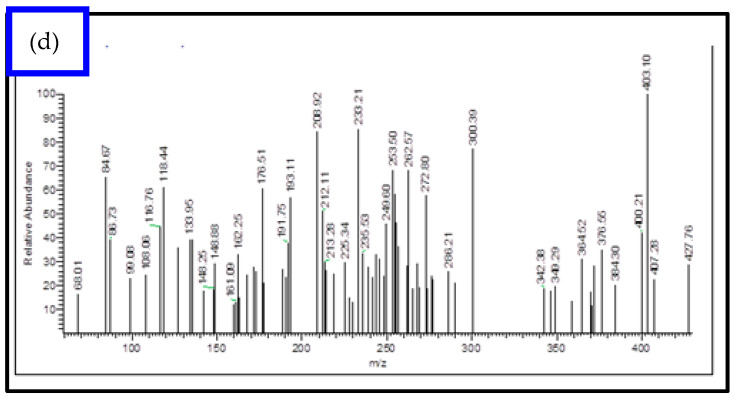
EI-mass spectral analysis of (**a**) [Cu(L)(NO_3_)(H_2_O)_3_], (**b**) [Zn(L)(NO_3_)(H_2_O)_3_], (**c**) [La(L)(NO_3_)_2_(H_2_O)_2_], and (**d**) [VO(L)(OC_2_H_5_)(H_2_O)_2_] complexes.

ESI-MS spectra were utilized to examine the structure of both the Schiff base ligands and their metal complexes. By comparing the measured *m*/*z* values with the molecular formula weights, the proposed molecular formulas for these compounds were confirmed. In the mass spectrum of the ligand, a molecular ion peak at *m*/*z* 297.0 is observed, supporting the proposed formula. Furthermore, the Schiff base ligand exhibits a series of peaks at *m*/*z* 170.0, 144.0, 89.0, and 63.0, corresponding to various fragments as depicted in Scheme 1. This information strengthens the identification of the structures based on the proposed molecular formulas for the synthesized compounds. Similarly, the mass spectra of the [Cu(L)(NO_3_)(H_2_O)_3_], [Zn(L)(NO_3_)(H_2_O)_3_], [La(L)(NO_3_)_2_(H_2_O)_2_], [Cr(L)(NO_3_)_2_(H_2_O)_2_], and [VO(L)(OC_2_H_5_)(H_2_O)_2_] complexes reveal the presence of their respective parent molecular ions, with *m/z* values of 488.72, 472.17, 595.57, 446.00, and 427.76, respectively, confirming their molecular weights (Figure 2 and Figure S2). The available mass spectral data collectively confirm the successful synthesis of the Schiff base ligand and its associated metal complexes. Both the ligand and its complexes exhibit molecular ion peaks and associated fragmentation peaks in their mass spectra. The construction of the structures of the metal complexes is feasible through the interpretation of elemental analyses, spectroscopic data (including infrared, electronic, ^1^HNMR, and mass spectroscopy), and magnetic susceptibility measurements. Scheme 2 illustrates the proposed structures of the metal complexes [20,22].

### 2.4. Electronic Spectra

The newly synthesized compounds exhibit specific structural geometries as determined by their electronic spectral data and magnetic moment values [23]. Electronic spectroscopy was employed to verify the stereochemistry of the metal ions in the complexes, considering the position of the metal ions and the number of transition peaks. The corresponding spectra can be found in Figure S3, and Table 3 provides the maximum absorption wavelength (λ_max_) values along with their assignments. Electronic absorption spectra were obtained for HL and its complexes in the wavelength range of 200–800 nm at a temperature of 298 K. The π→ π* and n → π* transitions of HL, associated with the ligand’s azomethine group, were observed at λ_max_ 287 nm and λ_max_ = 334 nm, respectively. Upon complex formation, the n → π* transition shifted to higher wavelengths, ranging from 353 to 360 nm, indicating the involvement of the azomethine group in the coordination process. Ligand-to-metal charge transfer bands were observed in the absorption spectra of [Zn(L)(NO_3_)(H_2_O)_3_], [VO(L)(OC_2_H_5_)(H_2_O)_2_], [Cr(L)(NO_3_)_2_(H_2_O)_2_], [Cu(L)(NO_3_)(H_2_O)_3_], and [La(L)(NO_3_)_2_(H_2_O)_2_] complexes at λ_max_ = 484, 486, 430, 488, and 490 nm, respectively. These bands confirm the octahedral geometry surrounding the [Zn(L)(NO_3_)(H_2_O)_3_], [Cr(L)(NO_3_)_2_(H_2_O)_2_], [Cu(L)(NO_3_)(H_2_O)_3_], and [La(L)(NO_3_)_2_(H_2_O)_2_] metal ions [24,25]. The [Cu(L)(NO_3_)(H_2_O)_3_] complex exhibited a d-d transition band at 550 nm (^2^B_1g_ → ^2^E_g_), while the CrL complex displayed a band at 535 nm (^3^A_2g_(F) → ^3^T_2g_(F)), confirming the octahedral structure of both complexes. These complexes are paramagnetic due to the presence of an unpaired electron. The [VO(L)(OC_2_H_5_)(H_2_O)_2_] complex spectrum showed a wide band at 532 nm, attributed to the ^2^B_1g_ (d_z_^2^) → ^2^E_g_ (d_xz_, d_yz_) and ^2^T_2g_ → ^2^E_g_ transitions in the deformed square pyramidal geometry of the [VO(L)(OC_2_H_5_)(H_2_O)_2_] complex [26,27].

### 2.5. Powder XRD Studies

Powder X-ray diffraction (XRD) patterns were used to analyze the [Cu(L)(NO_3_)(H_2_O)_3_], [Cr(L)(NO_3_)_2_(H_2_O)_2_], [Zn(L)(NO_3_)(H_2_O)_3_], and [VO(L)(OC_2_H_5_)(H_2_O)_2_] metal complexes, as depicted in Figures S4 and S5. The patterns revealed distinct characteristics for each complex. The XRD patterns of [Cr(L)(NO_3_)_2_(H_2_O)_2_], [Zn(L)(NO_3_)(H_2_O)_3_], and [VO(L)(OC_2_H_5_)(H_2_O)_2_] exhibited a combination of polycrystalline and amorphous structures, which were represented by a broad peak spanning a range of 2θ = 5–80° [28,29]. This indicates the presence of both crystalline and non-crystalline components in these complexes. In contrast, the XRD pattern of the [Cu(L)(NO_3_)(H_2_O)_3_] complex displayed several intense peaks at specific angles. Notable peaks were observed at 2θ = 14.3°, 17.4°, 23.5°, 28.2°, 34.3°, 37.6°, 40.5°, 44.2°, and 47.3°, with the maximum intensity occurring at 2θ = 28.2°.The level of crystallinity of the [Cu(L)(NO_3_)(H_2_O)_3_], [Cr(L)(NO_3_)_2_(H_2_O)_2_], [Zn(L)(NO_3_)(H_2_O)_3_], and [VO(L)(OC_2_H_5_)(H_2_O)_2_] complexes was determined by analyzing their powder X-ray diffraction patterns. The average crystallite size (ξ) can be calculated using the XRD pattern and the Debye–Scherrer equation.

ξ = λβ(1)

In the equation, the reference peak width at the angle (θ) was utilized, together with the X-ray wavelength (1.5425 Å), K constant (chosen as 0.95 for organic substances), and β_1/2_ the width at half maximum of the reference diffraction peak measured in radians. The dislocation density was used to determine how many dislocation lines there were in each unit of crystallographic area. The average particle diameter was computed using the following formula to arrive at the value of (ξ) [9,10].

δ = ξ(2)

The synthesized HL ligand and the [Cu(L)(NO_3_)(H_2_O)_3_], [La(L)(NO_3_)_2_(H_2_O)_2_], and [VO(L)(OC_2_H_5_)(H_2_O)_2_] complexes were analyzed using a powder X-ray diffractometer with a Cu anode material, where the X-ray wavelength (Kα) was measured to be 1.5406 Å. The powder X-ray diffraction (XRD) patterns of the transition metal complexes, labeled as metal complexes 1 and 2, revealed a combination of polycrystalline and amorphous structures. This was observed as a broad peak spanning a range of 2θ = 5–80° [28,29].

### 2.6. The pH Stability Range of the New Complexes

**Figure 3:**
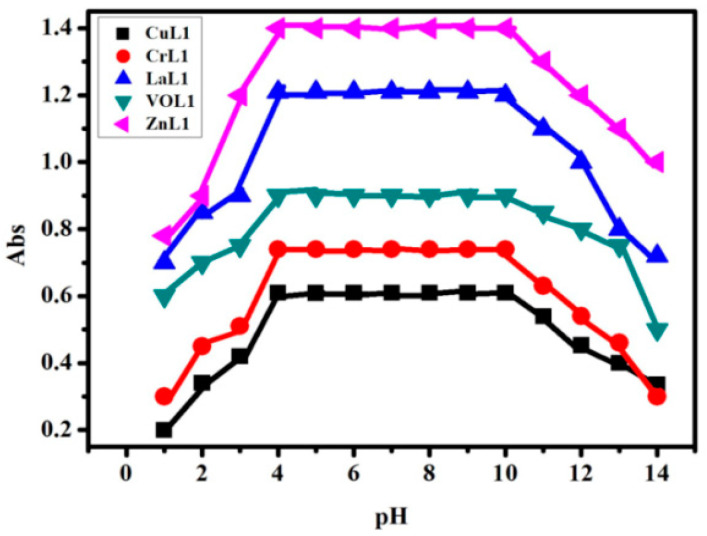
pH stability profile of the [Cu(L)(NO_3_)(H_2_O)_3_], [Cr(L)(NO_3_)_2_(H_2_O)_2_], [La(L)(NO_3_)_2_(H_2_O)_2_], [VO(L)(OC_2_H_5_)(H_2_O)_2_], and [Zn(L)(NO_3_)(H_2_O)_3_], complexes in DMF at different pH values.

The pH profile of the tested complexes exhibits well-defined dissociation curves and demonstrates excellent stability across a wide pH range of 4 to 11. This behavior indicates that the presence of our new HL ligand enhances the stability of all the synthesized complexes. Consequently, the pH range between 4 and 11 is deemed suitable for various applications involving these compounds. Moreover, the obtained results indicate that the synthesized complexes exhibit significantly higher stability compared to their corresponding ligands [30] (Figure 3).

### 2.7. DFT Study

**Figure 4:**
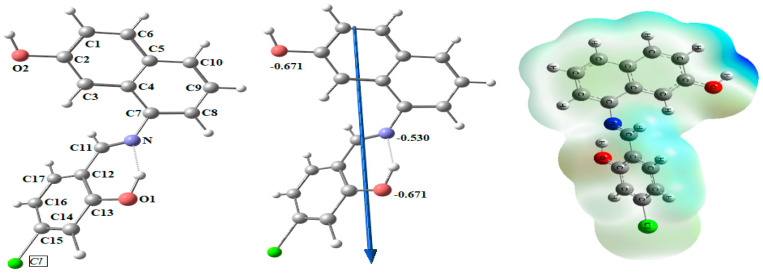
Natural charges on atoms, the molecular electrostatic potential (MEP) surface by density function B3LYP/6-311++g(d, p), the vector of the dipole moment, and the optimal ligand structure.

Molecular DFT calculation of ligand (HL):

Figure 4 shows the optimized structures of the ligand as the lowest energy configurations. The natural charges obtained from Natural Bond Orbital Analysis (NBO) show that the more negative active sites are O1 (−0.671), O2 (−0.671), and N (−0.530). So, the ligand is coordinated with metal ions as a bidentate, forming a six-membered ring with O1 and N atoms.

**Figure 5:**
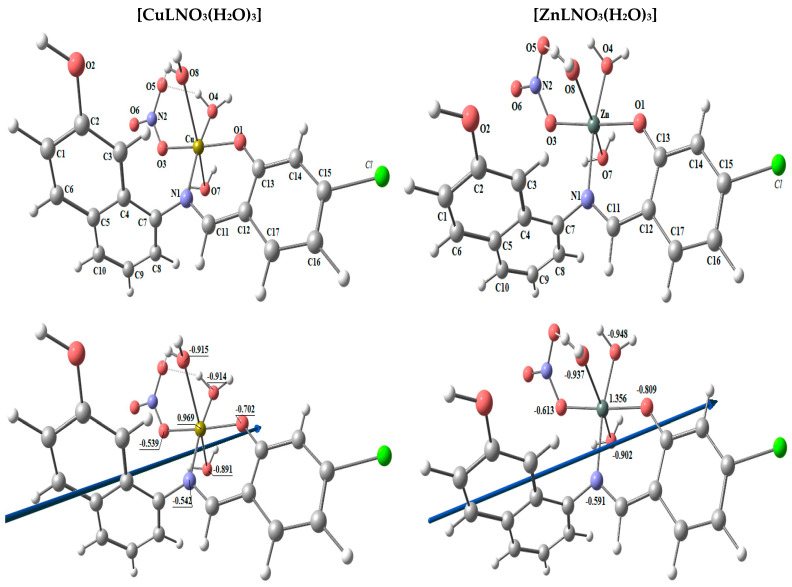

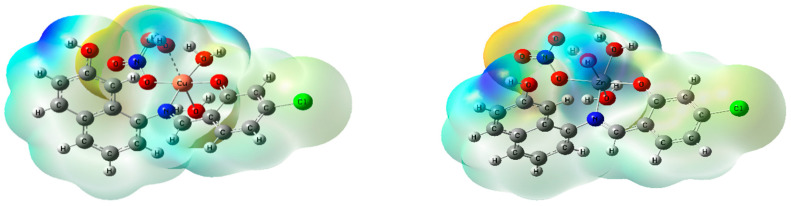
The optimized structure, the vector of the dipole moment, the natural charges on atoms, and the molecular electrostatic potential (MEP) surface on active centers of [CuLNO_3_(H_2_O)_3_] and [ZnLNO_3_(H_2_O)_3_] complexes.

Molecular DFT calculation of [CuLNO_3_(H_2_O)_3_] and [ZnLNO_3_(H_2_O)_3_] complexes:

The lowest energy configurations of the complexes [CuLNO_3_(H_2_O)_3_] and [ZnLNO_3_(H_2_O)_3_] are shown in Figure 5 as their optimized structures. In octahedral geometries, the copper and zinc atoms are six-coordinated; nevertheless, the atoms O1, N1, O3, and O4 are practically in one plane but are off by 3.620° and 3.698°, respectively. According to Table 4, the axial angles are almost linear with slight variations, and the tetrahedral structure angles are close to 90 degrees.

Using the NBO-analysis on the coordinated atoms, the natural charges calculated for [CuLNO_3_(H_2_O)_3_] and [ZnLNO_3_(H_2_O)_3_] are [Cu (+0.969), N1 (−0.542), O1 (−0.702), O3 (−0.539), O4 (−0.914), O7 (−0.891), and O8 (−0.915)] and [Zn (+1.356), N1 (−0.591), O1 (−0.809), O3 (−0.613), O4 (−0.948), O7 (−0.902), and O8 (−0.937)], respectively [3].

**Figure 6:**
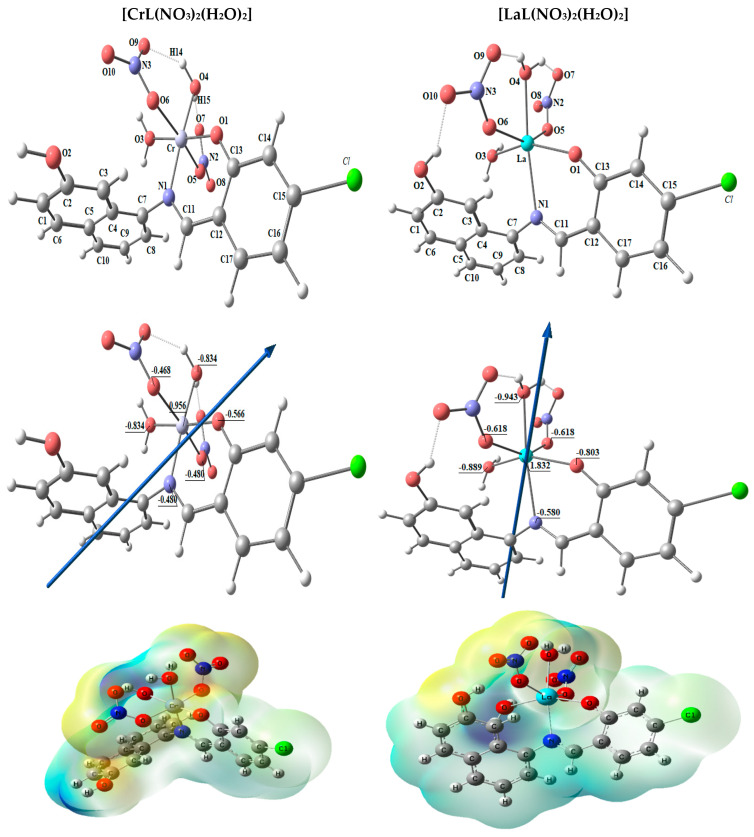
The molecular electrostatic potential (MEP) surface on active centers of the optimized structure of [CrL(NO_3_)_2_(H_2_O)_2_] and [LaL(NO_3_)_2_(H_2_O)_2_] complexes, the dipole moment’s vector, the atoms’ natural charges.

Molecular DFT calculation of [CrL(NO_3_)_2_(H_2_O)_2_] and [LaL(NO_3_)_2_(H_2_O)_2_] complexes:

The complexes [CrL(NO_3_)_2_(H_2_O)_2_] and [LaL(NO_3_)_2_(H_2_O)_2_] have been tuned to have the lowest energy configurations, which are shown in Figure 6 as their optimized structures. In octahedral geometries, the chromium and lanthanum atoms are six-coordinated; nevertheless, the atoms O1, N1, O3, and O4 are practically in one plane but are off by 2.692° and −7.102°, respectively. The axial angles are almost linear with slight variations and the angles in the tetrahedral shape are close to 90 degrees, according to Table 5. The natural charges computed from the NBO-analysis on the coordinated atoms for [CrL(NO_3_)_2_(H_2_O)_2_] and [LaL(NO_3_)_2_(H_2_O)_2_] are [Cr (+0.956), N1 (−0.480), O1 (−0.566), O3 (−0.834), O4 (−0.834), O5 (−0.480), and O6 (−0.468)] and [La (+1.832), N1 (−0.580), O1 (−0.803), O3 (−0.889), O4 (−0.943), O5 (−0.803), and O6 (−0.618)], respectively.

**Figure 7:**
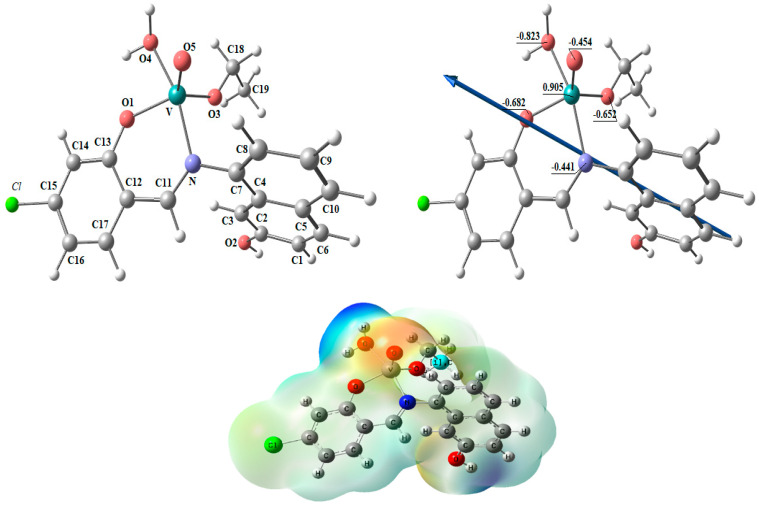
The optimized structure, the vector of the dipole moment, the natural charges on atoms, and the molecular electrostatic potential (MEP) surface on active centers of [VOL(H_2_O)OEt] complex.

Molecular DFT calculation of [VOL(H_2_O)OEt] complex:

Figure 7 shows the optimized structures of the complex [VOL(H_2_O)OEt] as the lowest energy configurations. The vanadium atom is five-coordinated in distorted square pyramidal geometry. The significant bond lengths and angles are cited in Table 6. For [VOL(H_2_O)OEt], the coordinated atoms’ natural charges from the NBO analysis are V (+0.905), N (−0.441), O1 (−0.682), O3 (−0.652), O4 (−0.823), and O5 (−0.454).

**Figure 8:**
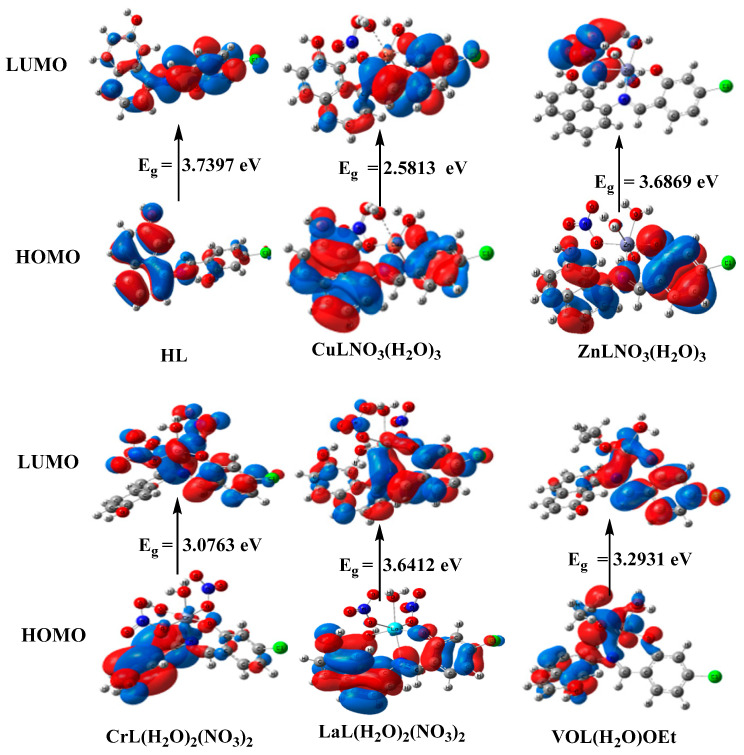
HOMO and LUMO charge density maps of HL, [CuLNO_3_(H_2_O)_3_], [ZnLNO_3_(H_2_O)_3_], [CrL(NO_3_)_2_(H_2_O)_2_], [LaL(NO_3_)_2_(H_2_O)_2_], and [VOL(H_2_O)OEt].

For the ligands and complexes, the computed total energy, the highest occupied molecular orbital (HOMO) energies, the lowest unoccupied molecular orbital (LUMO) energies, and the dipole moment were determined, as shown in Table 7. The fact that the total energy of the complexes is more negative than the total energy of the free ligand suggests that the complexes are more stable than the free ligands and that the energy gap (Eg) = ELUMO − EHOMO is lower for the complexes than for the ligand because of chelation of the ligand to metal ions, as shown in Table 4 [4,11]. Upon compound formation, Figure 8’s charge transfer interactions are explained by the lowering of Eg in complexes relative to that of the ligand. It has been suggested that the HOMO and LUMO energies may be used to calculate the ionization potential (I), electron affinity (A), electronegativity (E), chemical potential (P), hardness (H), softness (S), and electrophilicity index (E), which are all used to understand different aspects of reactivity related to chemical processes Table 7.

### 2.8. Microbiological Studies

#### Antibacterial Study

**Figure 9:**
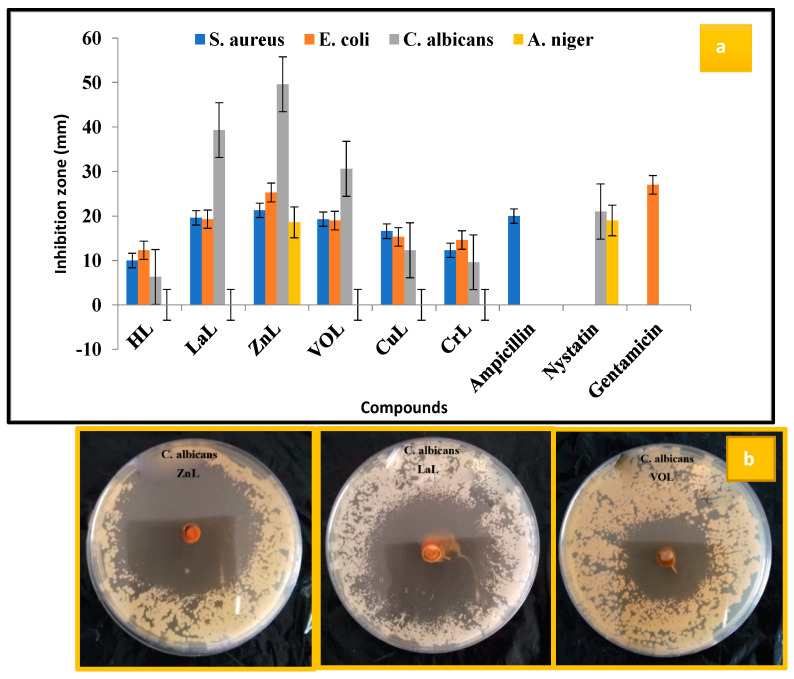
(**a**) Graphical representation of antimicrobial activity results of HL ligand and its new [Cu(L)(NO_3_)(H_2_O)_3_], [Zn(L)(NO_3_)(H_2_O)_3_], [VO(L)(OC_2_H_5_)(H_2_O)_2_], [Cr(L)(NO_3_)_2_(H_2_O)_2_], and [La(L)(NO_3_)_2_(H_2_O)_2_] complexes at 20 µg/mL concentration and (**b**) zone of inhibition of [Zn(L)(NO_3_)(H_2_O)_3_], [La(L)(NO_3_)_2_(H_2_O)_2_], and [VO(L)(OC_2_H_5_)(H_2_O)_2_] against *C. albicans*.

To assess the antibacterial activity of the ligand and its metal complexes and develop effective inhibitors with a unique mode of action, various strains of bacteria and fungi were employed as test subjects. The antimicrobial activity of the HL ligand and its novel complexes, namely [Cu(L)(NO_3_)(H_2_O)_3_], [Zn(L)(NO_3_)(H_2_O)_3_], [VO(L)(OC_2_H_5_)(H_2_O)_2_], [Cr(L)(NO_3_)_2_(H_2_O)_2_], and [La(L)(NO_3_)_2_(H_2_O)_2_], was evaluated. Comparative analysis of the data revealed that the [Cu(L)(NO_3_)(H_2_O)_3_], [Zn(L)(NO_3_)(H_2_O)_3_], [VO(L)(OC_2_H_5_)(H_2_O)_2_], [Cr(L)(NO_3_)_2_(H_2_O)_2_], and [La(L)(NO_3_)_2_(H_2_O)_2_] complexes exhibited enhanced activity compared to the free HL ligand. Overton’s theory was employed to elucidate the rationale behind the heightened efficacy of the complexes. The antimicrobial efficiency of the newly synthesized compounds was assessed by measuring the inhibition zone, as depicted in Figure 9 and Figure S7, Table S2. The activity of the complexes followed the order: [Zn(L)(NO_3_)(H_2_O)_3_] > [La(L)(NO_3_)_2_(H_2_O)_2_] > [VO(L)(OC_2_H_5_)(H_2_O)_2_] > [Cu(L)(NO_3_)(H_2_O)_3_] > [Cr(L)(NO_3_)_2_(H_2_O)_2_] > HL. Notably, the [Cr(L)(NO_3_)_2_(H_2_O)_2_] complex displayed no activity against the tested strains. Conversely, the [Zn(L)(NO_3_)(H_2_O)_3_] and [La(L)(NO_3_)_2_(H_2_O)_2_] complexes demonstrated remarkable fungicidal activity against A. Niger and *C. albicans*, exhibiting inhibition zones three times wider than those observed with the Nystatin antibiotic [12,13,14,31,32]. Our findings agree well with the antimicrobial results of similar bidentate Schiff bases [33,34,35,36,37].

### 2.9. DNA Binding Study

#### 2.9.1. Absorption Titration

**Figure 10:**
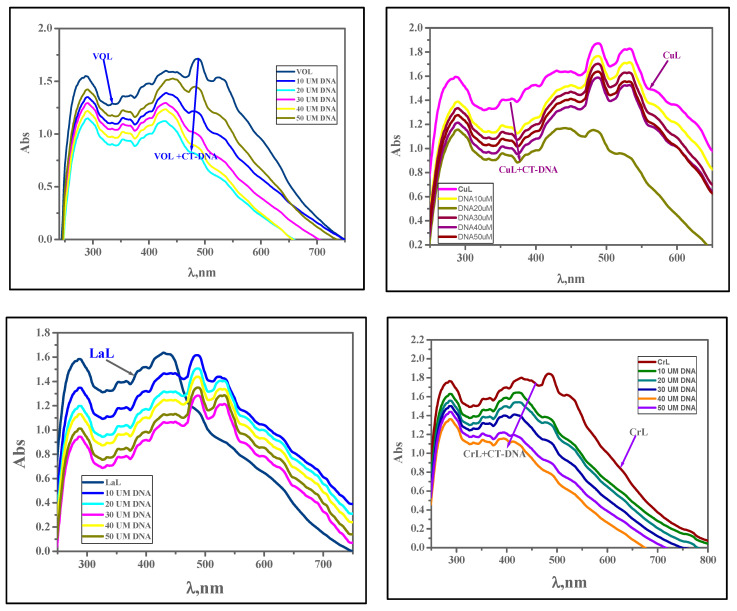

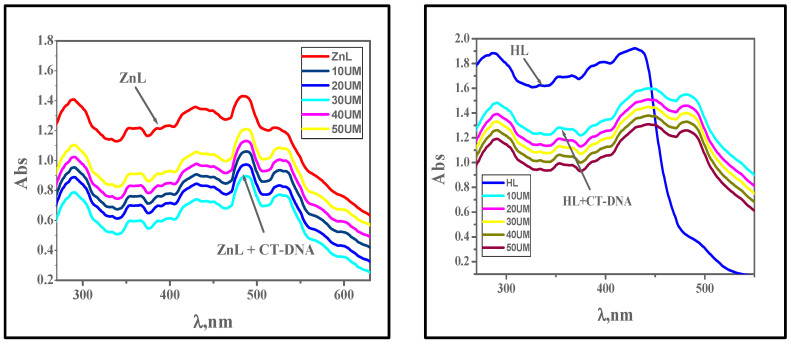
Absorption spectra of the complexes [VO(L)(OC_2_H_5_)(H_2_O)_2_], [Cu(L)(NO_3_)(H_2_O)_3_], [La(L)(NO_3_)_2_(H_2_O)_2_], [Cr(L)(NO_3_)_2_(H_2_O)_2_], [Zn(L)(NO_3_)(H_2_O)_3_], and HL in the presence of increasing concentrations of CT-DNA (in Tris-HCl/NaCl buffer). With increasing concentrations of CT-DNA, the absorbance of the complex changes at [complex] = 10 μM and [DNA] = 10–100 μM.

By adding calf thymus DNA (10–50 μM) to the fixed complex concentration (10 μM), electronic absorption measurements were conducted to assess the amount of absorption. Figure 10 and Table 8 show the bathochromic and hypochromic shifts in the spectrum, and binding strength (K_b_) values were calculated using the study’s findings. When a complex interacts with DNA by intercalation, the DNA undergoes a structural change that results in its elongation and exhibits a shift in the bathochromic and hypochromic regions of its electronic spectra. DNA base pairs interact with the intercalated chromophore moiety of the metal complex by a process known as π–π stacking, which causes a hypochromic shift. Bathochromic effects result from the binding of the DNA base pair’s π–π* orbitals with the metal complexes, which lowers the π → π* transition energy. By calculating K_b_ values, the binding strengths of the complexes [Cu(L)(NO_3_)(H_2_O)_3_], [Zn(L)(NO_3_)(H_2_O)_3_], [VO(L)(OC_2_H_5_)(H_2_O)_2_], [Cr(L)(NO_3_)_2_(H_2_O)_2_], and [La(L)(NO_3_)_2_(H_2_O)_2_] with DNA were evaluated. The values were found to be 5.40 × 10^5^, 7.30 × 10^5^, 6.00 × 10^5^, 4.40 × 10^5^, and 6.70 × 10^5^ M^−1^, respectively. This indicates strong DNA binding and is equivalent to the value of ethidium bromide, which is 10^7^ M^−1^. According to the absorption experiments, [Zn(L)(NO_3_)(H_2_O)_3_] > [La(L)(NO_3_)_2_(H_2_O)_2_] > [VO(L)(OC_2_H_5_)(H_2_O)_2_] > [Cu(L)(NO_3_)(H_2_O)_3_] > [Cr(L)(NO_3_)_2_(H_2_O)_2_] > HL was the order in which the complexes’ binding strengths were displayed [15]. Comparing the K_b_ values of our compounds [Cr(L)(NO_3_)_2_(H_2_O)_2_] and [Cu(L)(NO_3_)(H_2_O)_3_], which were obtained from the chloro-substituted Schiff base with similar complexes of the methoxy-substituted Schiff base [38], one can observe that our compounds have stronger interaction to CT-DNA according to the K_b_ values of our compounds 3.97 × 10^5^  and 6.70 × 10^5^ M^−1^ for [Cr(L)(NO_3_)_2_(H_2_O)_2_] and [Cu(L)(NO_3_)(H_2_O)_3_], respectively. As opposed to this, the methoxy-substituted Schiff base’s Cr(III) and Cu(II) complexes exhibit K_b_ values of 4.40 × 10^4^  and 6.74 × 10^4^ M^−1^, respectively.

#### 2.9.2. Viscosity Studies

**Figure 11:**
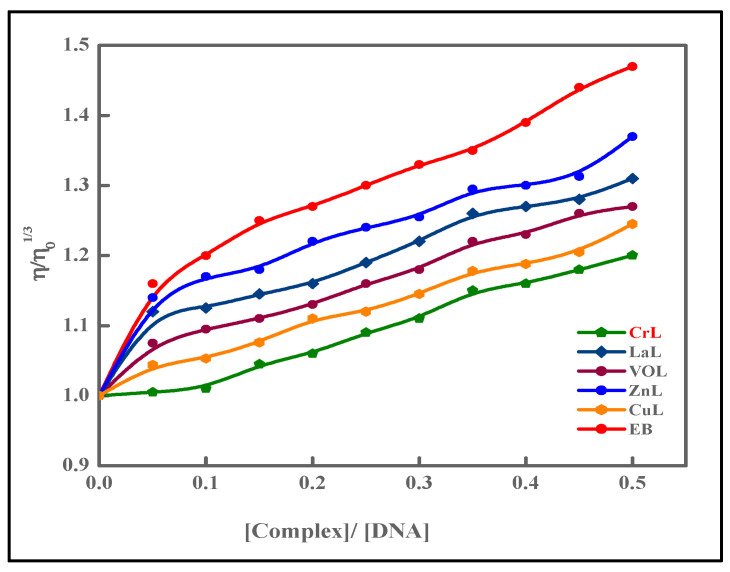
The relative viscosity of CT-DNA is affected by the quantity of ethidium bromide (EB) and the metal complexes.

Viscosity experiments provided additional support for DNA interaction investigations with metal complexes. DNA’s double helix structure lengthens because of the intercalation of a drug moiety, increasing viscosity. Viscosity rises because of the experiment’s intercalative method of interaction between ethidium bromide and DNA [16]. In the current investigation, metal complexes were introduced in increments [0–120 μM] to the fixed concentration of calf thymus DNA [10 μM], and the rise in viscosity was comparable to that of ethidium bromide. The order is given as EB > [Zn(L)(NO_3_)(H_2_O)_3_] > [La(L)(NO_3_)_2_(H_2_O)_2_] > [VO(L)(OC_2_H_5_)(H_2_O)_2_] > [Cu(L)(NO_3_)(H_2_O)_3_] > [Cr(L)(NO_3_)_2_(H_2_O)_2_] > HL Figure 11. The interaction of metal complexes with calf thymus DNA by intercalation is therefore further supported by viscosity.

#### 2.9.3. Gel Electrophoresis

**Figure 12:**
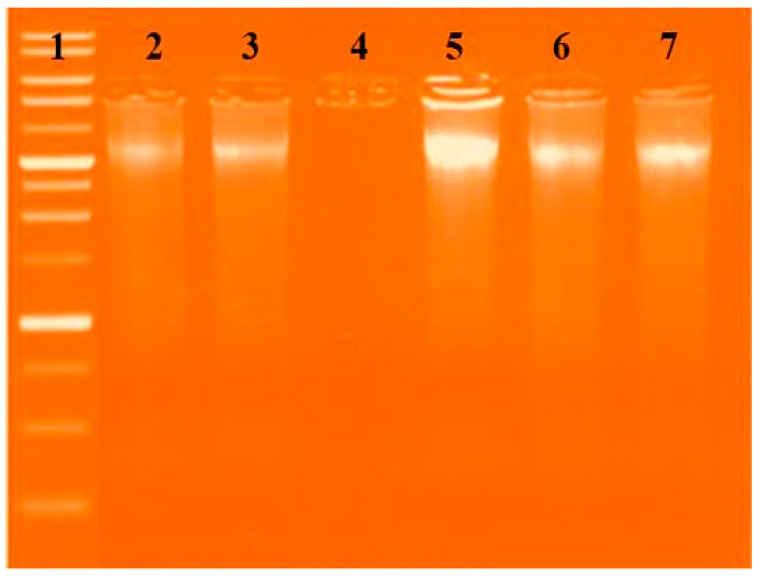
Gel electrophoresis pattern showing the interactions of the new complexes with DNA based on gel electrophoresis. Lane 1: DNA Ladder, Lane 2: HL + DNA, Lane 3: [Cu(L)(NO_3_)(H_2_O)_3_]  +  DNA, Lane 4: [VO(L)(OC_2_H_5_)(H_2_O)_2_] +  DNA, Lane 5: [La(L)(NO_3_)_2_(H_2_O)_2_] + DNA, and Lane 6: [Zn(L)(NO_3_)(H_2_O)_3_]  +  DNA, Lane 7: [CrL(NO3)2(H2O)2] + DNA.

The definitive method to investigate how CT-DNA interacts with the new complexes is agarose gel electrophoresis. The gel electrophoresis picture showed that the CT-DNA loaded with the new complexes had a somewhat decreased intensity in the CuL complex and disappeared in the LaL complex due to the complete cleavage for CT-DNA Figure 12 [3,12,13,14,39,40,41].

### 2.10. Free Radical Scavenging Activity

**Figure 13:**
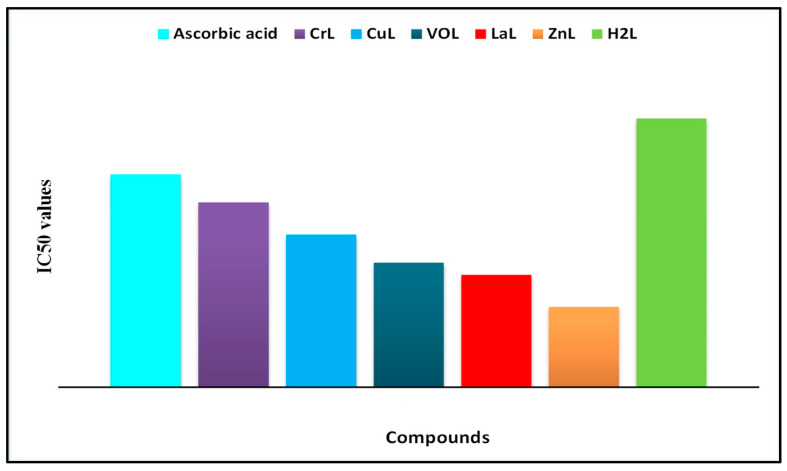
Concentration-dependent IC_50_ values of metal complexes’ DPPH radical-scavenging activity.

Reactive oxygen species (ROS), including hydrogen peroxide, superoxide anion, and hydroxyl radical, are highly reactive and potentially harmful chemical agents generated during metabolic processes in the human body. They are known to cause oxidative damage, leading to various illnesses such as diabetes, cancer, Alzheimer’s disease, coronary heart disease, and Parkinson’s disease, affecting proteins, nucleic acids, and lipids. Consequently, it is crucial to protect our bodies from oxidative stress by administering antioxidant-rich drugs that can slow down or prevent this process. In the antioxidant assessment of all synthesized metal complexes, ascorbic acid was employed as a control at different doses using the DPPH radical. Both the metal complexes and the standard exhibited a dose-dependent increase in antioxidant activity. Figure 13 illustrates the dose–response curves representing the ability of the metal complexes to scavenge DPPH radicals. Furthermore, all metal complexes demonstrated potent scavenging activity, with the lowest IC_50_ values. The order of IC_50_ values for antioxidant activity among the complexes and reference drugs was found to be [Zn(L)(NO_3_)(H_2_O)_3_] > [La(L)(NO_3_)_2_(H_2_O)_2_] > [VO(L)(OC_2_H_5_)(H_2_O)_2_] > [Cu(L)(NO_3_)(H_2_O)_3_] > [Cr(L)(NO_3_)_2_(H_2_O)_2_] > HL. This can be attributed to the ability of the samples to disrupt the free radical cascade by providing a hydrogen atom. Therefore, the study results indicate that all complexes possess significant antioxidant activity, with the [Zn(L)(NO_3_)(H_2_O)_3_] complex being the most efficient, displaying the lowest IC_50_ value, and holding promise as a potential treatment for degenerative diseases resulting from oxidative stress. Consequently, these complexes have substantial potential as free radical scavengers [32,42].

### 2.11. In Vitro Anti-Inflammatory Activity

Inflammation is a biological defense response that can be triggered by various stressors such as heat, radiation, microbial infections, chemical agents, or physical agents, often accompanied by pain. Prostaglandins, metabolized through the arachidonic acid pathway via the cyclooxygenase-2 (COX-2) enzyme cascade, play a significant role in the inflammation process. Inhibition of COX-2 can directly reduce prostaglandin production, as these hormones contribute to the body’s response to painful stimuli. Consequently, a medication capable of inhibiting these enzymes could possess anti-inflammatory and analgesic properties.

**Figure 14:**
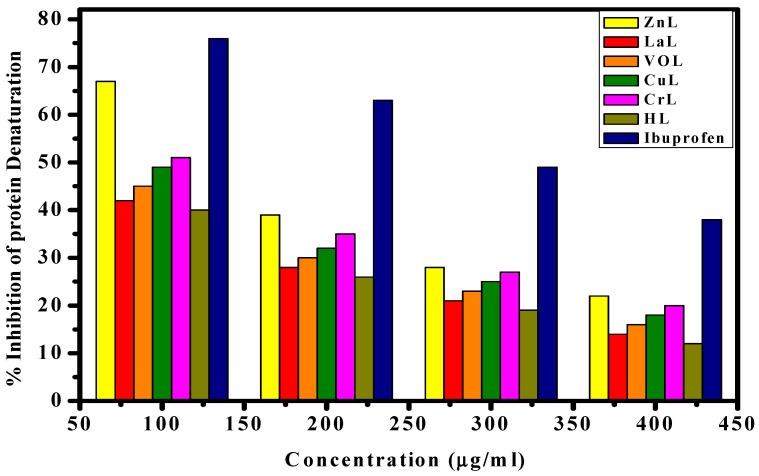
Inhibition % of protein denaturation of HL ligand and its metal complexes compared to ibuprofen at different concentrations.

To compare the anti-inflammatory properties of the novel compounds with the standard medication ibuprofen, the percentage suppression of protein denaturation in fresh egg albumin was measured. The results, presented in terms of IC_50_ values, are depicted in Figure 14. The anti-inflammatory findings revealed that the inhibition of protein denaturation increased with concentration. The order of protein denaturation inhibition among the complexes was as follows: [Zn(L)(NO_3_)(H_2_O)_3_] > [La(L)(NO_3_)_2_(H_2_O)_2_] > [VO(L)(OC_2_H_5_)(H_2_O)_2_] > [Cu(L)(NO_3_)(H_2_O)_3_] > [Cr(L)(NO_3_)_2_(H_2_O)_2_] > HL. Notably, the [Zn(L)(NO_3_)(H_2_O)_3_] complex exhibited the highest effectiveness as an anti-inflammatory metal complex, with an IC_50_ value of 2.5 (2) M, suggesting its potential application in the treatment of inflammation-related disorders [43,44].

### 2.12. Molecular Docking Study

**Figure 15:**
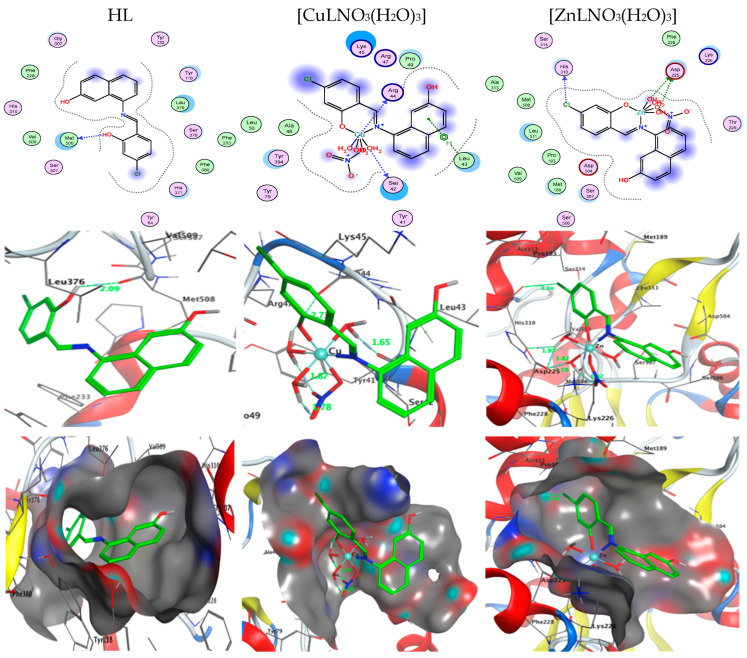
Two-dimensional and three-dimensional plots of the interactions between HL, [CuLNO_3_(H_2_O)_3_], and [ZnLNO_3_(H_2_O)_3_] with the active site of the receptor of *Candida albicans* (PDB ID: PDB ID: 5V5Z). Hydrophobic interactions with amino acid residues are shown with dotted curves.

**Figure 16:**
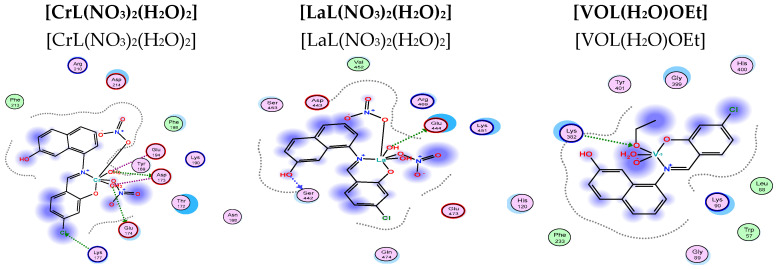

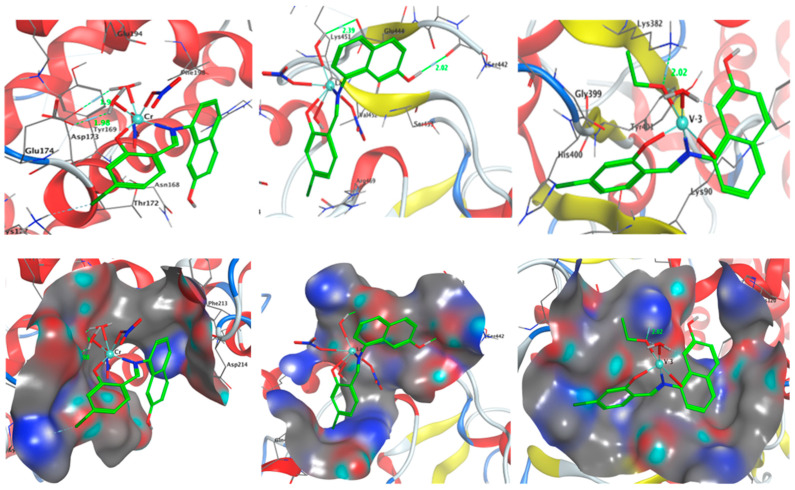
Two-dimensional and three-dimensional plots of the interactions between [CrL(NO_3_)_2_(H_2_O)_2_], [LaL(NO_3_)_2_(H_2_O)_2_], and [VOL(H_2_O)OEt] with the active site of the receptor of *Candida albicans* (PDB ID: PDB ID: 5V5Z). Hydrophobic interactions with amino acid residues are shown with dotted curves.

A potent method for examining the biological activity of target molecules and producing unique structural characteristics for the creation of novel therapies is molecular docking. Additionally, it is a common computational technique for determining binding sites with appropriate conformations and estimating binding affinity. This is a useful way to discover a molecule’s ideal alignments and binding characteristics to create a new complex with the least amount of energy. Three-dimensional and two-dimensional docking architectures are shown, respectively, in Figure 15 and Figure 16. Additionally, computer docking tests were employed to determine how well these medicines were bound [26,27,45,46,47].

Docking on *Candida albicans* oxidoreductase/oxidoreducatse inhibitor (PDB ID: 5V5Z).

The binding free energy of ligand and complex with the receptor of *Candida albicans* OXIDOREDUCTASE/OXIDOREDUCATSE INHIBITOR (PDB ID: PDB ID: 5V5Z) are found to be −1.5, −28.7, −58.0, −53.0, −5.0, and −21.5 kcal/mol for HL, [CuL(NO_3_)(H_2_O)_3_], [ZnLNO_3_(H_2_O)_3_], [CrL(NO_3_)_2_(H_2_O)_2_], [LaL(NO_3_)_2_(H_2_O)_2_], and [VOL(H_2_O)OEt], respectively. Table 9 showed the presence of effective van der Waal interactions and hydrogen bonding at the distance from 2.60–3.27 Å at the binding sites O34, O35, O44, Cl22 and the amino acid residues ASP225, ARG44, GLU174, and LYS 177. According to the scoring values, the greater the contact, the greater negative the binding energy. Consequently, the interactions are listed in the following order: HL < [LaL(NO_3_)_2_(H_2_O)_2_] < [VOL(H_2_O)OEt] < [CuLNO_3_(H_2_O)_3_] < [CrL(NO_3_)_2_(H_2_O)_2_] < [ZnLNO_3_(H_2_O)_3_]. It is worth mentioning that the presence of the chloro side group of the new Schiff base ligand has affected the hydrogen donor and acceptor interactions as well as the presence of the nitrate group inside the coordination sphere of the complexes.

This sequence of binding energy scores revealed outstanding interaction of [ZnL(NO_3_)(H_2_O)_3_] complex with the receptor of *Candida albicans* OXIDOREDUCTASE, which agrees well with the experimental results and predicts a promising fungicidal potency against candida Albicans and could help in solving the problem of antibiotic resistance [33,34].

The 2D and 3D plots of the interactions of HL, [CuLNO_3_(H_2_O)_3_], [ZnLNO_3_(H_2_O)_3_], [CrL(NO_3_)_2_(H_2_O)_2_], [LaL(NO_3_)_2_(H_2_O)_2_], and [VOL(H_2_O)OEt] with the active site of the receptor of *Candida albicans* (PDB ID: PDB ID: 5V5Z) are shown in Figure 15 and Figure 16 [23,38].

### 2.13. Photocatalytic Degradation of MB

**Figure 17:**
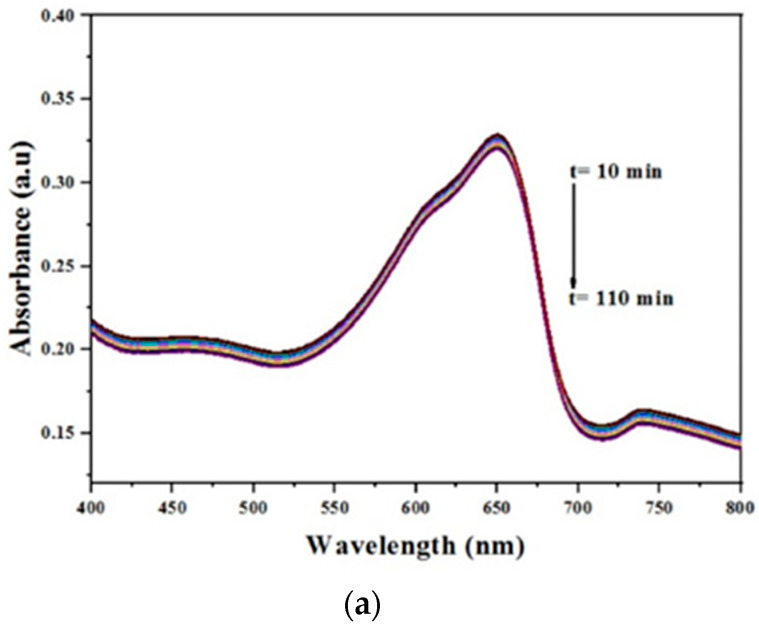

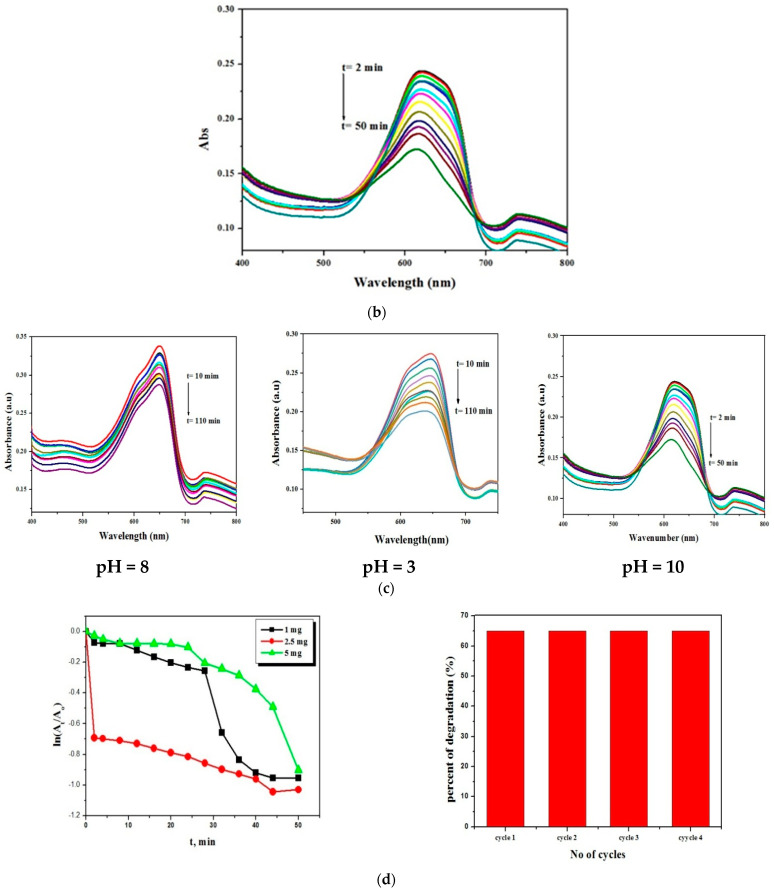
(**a**) The effect of Cu(II) Schiff base complex on MB photocatalytic degradation, [complex] = 2.5 mg, 30 mL of MB. (**b**) The temporal absorption spectrum changes of MB taking place under visible light irradiation for CuMGO catalyst, initial concentration of MB: 6.25 × 10^−3^ M, 30 mL, CuMGO: 2.5 mg and pH: 10. (**c**) Optimization of the pH for the degradation of MB. (**d**) Optimization of the amount of catalyst for degradation and (**b**) the recyclability of the catalyst.

The photocatalytic activity of the prepared catalyst was measured by the decrease in the intensity of the peak at 652 nm, corresponding to MB. Figure 17a shows the effect of the presence of the Cu(II) Schiff base complex before immobilization on the degradation of MB. The prepared complex has no efficiency in the degradation process.

The photocatalytic effect was studied in the presence of Cu(II) Schiff base complex/GO. The results revealed that under visible irradiation, the prepared complex could activate hydrogen peroxide for degradation of methylene blue within 50 min with a percent of conversion of 65%.

This was detected from the reduction of the maximum absorbance of MB; see Figure 17b. In addition, the characteristic peak of MB undergoes a hypsochromic shift to 616 nm, which could be attributed to the formation of N–de-ethylated intermediates. The rate of reaction has been calculated using the following Equation (3):

d C_t_/d_t_ = −K_app_ C_t_  or   ln(C_t_/C_o_) or ln(A_t_/A_o_) = −K_app_ t (3)

where C_0_ is the initial concentration of MB, C_t_ is the concentration at time ‘t’, A_o_ is the absorbance at t = 0, A_t_ the absorbance at time ‘t’, and K_app_ is the apparent rate constant, which can be calculated from the reduction of the peak intensity at 652 nm with time. The K_app_ was found to be 1.2 × 10^−2^.

#### 2.13.1. Effect of pH on the Degradation of MB

To study the effect of pH on the degradation process, the reaction was performed at different values of pH (3, 8, and 10) as shown in Figure 17c. From the obtained results, it is obvious that the catalytic reaction was fast and active in the basic medium and the degradation reaction took place in about 50 min, whereas in the neutral and acidic medium, it continued for about 110 min with 58.8% and 42.38%, respectively. This can be attributed to the increase in the concentration of OH^−^, which leads to an increased generation of OH^.^ and rapid degradation of MB.

#### 2.13.2. Effect of the Amount of Catalyst and Recyclability

The catalytic degradation reaction was recorded by keeping the concentration of MB constant at 6.25 × 10^−3^ M and varying the amounts of the catalyst (1 mg, 2.5 mg, and 5 mg). Figure 17d shows that the degradation activity increases up to 2.5 mg, above which it shows a reduction in the degradation activity. This can be explained on the basis that by increasing the number of Cu(II) Schiff base complex/GO, the molecules available in the substrate are not adequate for adsorption and the additional amount of the catalyst is not involved in catalyst activity. For investigating the recyclability of the catalyst after the first experiment, the solid catalyst was filtered, washed with deionized water, and used for other consecutive experiments. It has been found that the prepared catalyst can be used for three other cycles with no loss of its activity; see Figure 17d.

#### 2.13.3. Photocatalytic Mechanism

**Figure 18:**
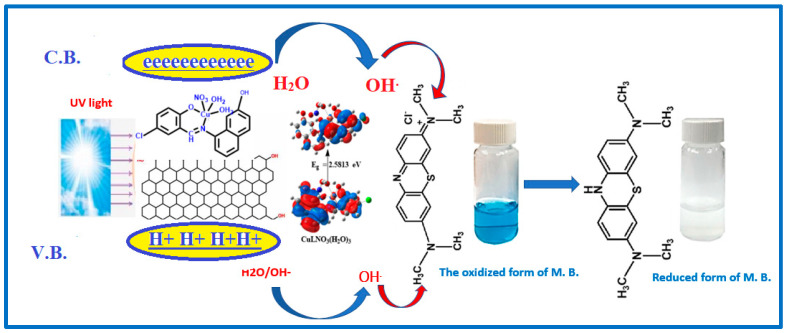
The possible photocatalytic mechanism for the degradation of MB in the presence of Cu(II) Schiff base complex loaded on graphene oxide and H_2_O_2_.

Based on the literature, the possible mechanism of photocatalytic degradation of MB can be described as follows [48,49]. When the photocatalyst [CuLNO_3_(H_2_O)_3_] is exposed to UV irradiation, an electron is transferred from the highest occupied molecular orbital (HOMO) of the oxygen and nitrogen atoms to the lowest unoccupied molecular orbital (LUMO) of the Cu(II) center. The HOMO strongly needs an electron to return to its stable state. As a result, an electron is captured from a water molecule, leading to the generation of hydroxyl (% OH) radicals. These active % OH radicals act as potent oxidizing agents, effectively breaking down the MB molecules and forming the final oxidation products, as depicted in Figure 18.

## 3. Experimental

### 3.1. Chemicals

All substances (including Zn(NO_3_)_2_.6H_2_O, VO(acac)_2_, Cu(NO_3_)_2_·2H_2_O, La(NO_3_)_3_·6H_2_O, and Cr(NO_3_)_2_·9H_2_O)) are of the analytical reagent grade and must be used exactly as directed. Calf thymus DNA (CT-DNA), 5-chloro-salicylaldehyde, 8-amino-2-naphthol, and Tris [hydroxymethyl]-amino methane were supplied by Sigma Aldrich (St. Louis, MO, USA).

### 3.2. Physical Measurements

Carbon, hydrogen, and nitrogen microanalyses were determined using a CHNS-932 (LECO) Varo elemental analyzer. Fourier transform infrared (FT-IR) spectra were obtained using a Shimadzu FTIR spectrophotometer (model 8101) in the range of 400–4000 cm^−1^. ^1^H and ^13^C NMR spectra were recorded in DMSO-d_6_ using a Bruker 400 MHz spectrometer with TMS as an internal standard. Mass spectra were obtained using an electron ionization method at 70 eV on an HP MS-5988 GS-MS. UV-visible spectra were collected using a Shimadzu UVmini-1240 spectrophotometer. Molar conductivities of 10^−3^ M solutions of the solid complexes in DMF were measured using a Jenway 4010 conductivity meter. Differential thermogravimetric (DTG) and thermogravimetric measurements were performed on the solid complexes (TG).

### 3.3. Synthesis of Schiff Base Ligand

The HL ligand was synthesized by mixing 0.79 g of 8-amino-2-naphthol (5 mmol) with 0.783 g of 5-chlorosalicylaldehyde (5 mmol) in 30 mL of ethanol. The mixture was continuously stirred and refluxed for an hour at 80 °C with the addition of drops of acetic acid as a catalyst until a pale green precipitate formed, as shown in Scheme 2. The precipitate was recrystallized from ethanol, dried under vacuum over anhydrous CaCl_2_, and filtered. The obtained precipitate has the empirical formula C_1__7_H_1__2_NO_2_Cl, a melting point of 171 °C, solubility in ethanol, a yield percentage of 75%, and a molecular weight of 297.74 g/mol [9].

### 3.4. Synthesis of Schiff Base Metal Complexes

The five new complexes were prepared by refluxing equimolar amounts of Zn(NO_3_)_2_·6H_2_O (1 mmol, 0.297 g), La(NO_3_)_3_·6H_2_O (1 mmol, 0.325 g), VO(acac)_2_ (1 mmol, 0.265 g), Cu(NO_3_)_2_·2H_2_O (1 mmol, 0.188 g), and Cr(NO_3_)_2_·9H_2_O (1 mmol, 0.400 g) with 1 mmol (0.297 g) of the Schiff base ligand, as shown in Scheme 2. The reaction mixtures were homogenized using magnetic stirring for three hours at 60 °C. After 3 h, all complexes were separated, filtered, and rinsed with ethanol, their purity was checked using TLC, and then they were dried under a vacuum over anhydrous calcium chloride [3,9,10].

### 3.5. Antimicrobial Activity

The antimicrobial activity of the Schiff base ligand and its metal complexes was evaluated using the disc diffusion method. Different concentrations (1.0, 1.5 mg/mL) of the complexes were considered [15]. The selected cultures included gram-positive strains (*Staphylococcus aureus* ATCC: 13565), gram-negative strains (Escherichia coli ATCC: 10536), and fungal strains (*Candida albicans* ATCC: 10231 and *Aspergillus negar* ATCC: 16404).

For bacterial research, reference standards such as Gentamicin and Ampicillin, and for fungal investigations, Nystatin were used. Discs containing 5 mM concentrations of complexes and 1.0 mL of the microbial strain were placed on agar plates with the microbial strain and incubated for 24 h. The 5.5 × 10^5^ colonies of the selected microbial strain were then transferred to a nutrient broth medium, where they were left to grow. The observed zone of inhibition was compared with the standard drug to determine the antimicrobial activity [3,4,11].

### 3.6. DNA Interaction Studies

#### 3.6.1. Absorption Studies

UV-visible absorption titrations were conducted to investigate the DNA interaction of the new complexes. The complexes’ stock solution was prepared in DMSO as they were less soluble in the Tris-HCl buffer (pH = 7) solution. The complexes and DNA were maintained at concentrations of 10 and 0–50 µM, respectively. After adding a small amount of DNA solution to the complex solution, the solution was allowed to equilibrate for 5 min at 25 °C before recording the spectrum [12,15]. *K*_b_ values were calculated using the Wolfe–Shimmer formula:

[DNA]/*Ɛ*_a_ − *Ɛ*_f_ = [DNA]/*Ɛ*_b_ − *Ɛ*_f_ + 1/*K*_b_ [*Ɛ*_b_ − *Ɛ*_f_] (4)

where the concentration of DNA is given as [DNA], the apparent extinction coefficient is *Ɛ*_a_, the coefficient for the unbound metal complex is *Ɛ*_f_, and the extinction coefficient for the fully bound metal complex is *Ɛ*_b_. *K*_b_ was calculated using the slope-to-intercept ratio [5,6].

#### 3.6.2. Viscosity Studies

Viscosity experiments were carried out using Ostwald viscometer equipment at 37 ± 0.5 °C. The concentration of calf thymus DNA was fixed at 10 μM, while the complex concentration varied from 0 to 100 μM. Each experiment was performed twice for greater precision, and the flow time was averaged. The flow time was measured using a stopwatch. Based on the obtained data, a graph was plotted between (η/η_0_)^1/3^ and [complex]/[DNA], where η_0_ represents the viscosity of the DNA solution in the absence of the complex, and η signifies the viscosity in the presence of the complex [15,16].

#### 3.6.3. Gel Electrophoresis Studies

Gel electrophoresis was employed as the technique to assess how the tested complexes affect DNA. The novel complexes were dissolved in DMSO to prepare stock solutions with concentrations of 1 × 10^−3^ M. After incubating the mixtures of the complexes and CT-DNA for 1 h at 37 °C, 10 μL of each mixture was added to 4 μL of DNA loading buffer. The mixture was then injected into chamber wells pre-filled with TBE buffer and 1 percent agarose gel. The gel was run at 100 V in the electrophoresis tank for approximately 45 min. Photographs were taken using a Genius 3 Panasonic DMC-LZ5 Lumix camera to observe the movement of the samples in the agarose gel [39].

### 3.7. Free Radical Scavenging Activity

The antioxidant activity of the HL ligand and its complexes was evaluated spectrophotometrically using the 2,2-diphenyl-1-picrylhydrazyl (DPPH) free radical scavenging assay. A solution of DPPH in methanol (0.004 percent, *w/v*) was prepared and kept at 10 °C in the dark. Complexes with varied concentrations (1280, 640, 320, 160, 80, 40, 20, 10, and 0 µg/mL) were prepared in methanol. Each substance (40 mL) was added to 3 mL of the DPPH solution, and the absorbance was measured spectrophotometrically. The decrease in absorbance was monitored at 515 nm at intervals of 1 min until 16 min. The percentage inhibition (PI) of the DPPH radical was calculated using Equation (5). Ascorbic acid was used as a reference antioxidant for comparison [40].

PI = [(A_control_ − A_complex_)/A_control_] × 100% (5)

where A_complex_ represents the DPPH absorbance with the complex at t = 16 min, and A_control_ represents the DPPH absorbance of the radical with methanol at t = 0 min [7].

### 3.8. Anti-Inflammatory Activity

The in vitro anti-inflammatory impact of the Schiff base ligand and its metal complexes was evaluated using the egg albumin approach [18].

In a test tube, 0.2 mL of egg albumin, 2.8 mL of phosphate buffer, and 2 mL of each DMSO solution with concentrations ranging from 0 to 400 µM (pH 6.4) were added. The resulting solutions were incubated in a BOD at 37 °C for 30 min and then denatured by heating in a water bath at 70 °C for 20 min. After cooling the solution, the absorbance at 660 nm was measured. Equation (6) was used to calculate the percentage of protein denaturation inhibition and compare it with regular ibuprofen.

(6) $$\mathrm{inhibition}\;\%=\frac{A_{o}-A_{t}}{A_{o}}\times 100$$

where *A_t_* represents the sample’s absorbance, and *A_o_* represents the absorbance of the control sample. The IC_50_ value, defined as the concentration required to inhibit protein denaturation by 50% at its maximum level, was calculated for all samples.

### 3.9. Molecular Docking

Molecular docking investigations were performed using MOA2019 software. The binding modes of the most active site of oxidoreductase/oxidoreductase inhibitor (PDB ID: 5V5Z) were estimated. The optimized structure of HL and complexes, obtained from the calculations produced by Gaussian09, was saved in PDB file format. The crystal structures of the receptors were retrieved from the Protein Data Bank [41].

### 3.10. Photocatalytic Degradation of MB

The photocatalytic performance of the prepared complex was monitored under visible irradiation. In a typical experiment, 2.5 mg of [Cu(L)(NO_3_)(H_2_O)_3_] complex was mixed with 30 mL of MB (6.25 × 10^−3^ M) in a closed system and stirred magnetically for 30 min in the dark. The mixture was then radiated using a tungsten lamp (100 W), and 1 mL of H_2_O_2_ was added. At specific time intervals, 2 mL of the solution mixture was taken using a syringe, scanned with a UV-vis spectrophotometer, and the degradation of MB was quantitatively determined. After completing the catalytic reaction, the catalyst was filtered, washed with water, and dried. To confirm the activity of the prepared complex, it was used for three consecutive cycles under the same conditions [8]. The degradation efficiency was determined using Equation (7):

Degradation efficiency (%) = (C_0_ − C/C_0_) × 100 (7)

where C_0_ is the absorbance of MB at 652 nm after the adsorption–desorption equilibrium and C is the absorbance of MB after regular irradiation.

## 4. Conclusions

In conclusion, we successfully synthesized various metal complexes using the Schiff base ligand 8-[(1E)-(5-chloro-2-hydroxyphenyl) methylene] amino-2-naphthol. The structural characterization of both the Schiff base and its metal complexes was accomplished through the implementation of several analytical techniques, including magnetic susceptibility, UV-visible spectroscopy, FT-IR spectroscopy, and ESI-MS. The analytical studies confirmed a 1:1 stoichiometry for the metal complexes, which can be represented by the general formula [ML]. Based on the magnetic susceptibility, UV-vis spectral analyses, and DFT calculations, it was determined that all metal complexes exhibited octahedral geometry, except for the VO(II) complex, which displayed a square pyramidal shape. Moreover, the metal complexes demonstrated remarkable antioxidant potential as evidenced by their ability to scavenge the DPPH free radical and exhibit significant reduction power. Among the complexes tested, [ZnL(NO_3_)(H_2_O)_3_] exhibited the highest antioxidant potential, followed by [LaL(NO_3_)_2_(H_2_O)_2_], [VOL(H_2_O)OEt], [CuL(NO_3_)(H_2_O)_3_], [CrL(NO_3_)_2_(H_2_O)_2_], ascorbic, and HL. Notably, the [ZnL(NO_3_)(H_2_O)_3_] complex displayed particularly noteworthy antioxidant activity compared to the other synthesized metal complexes. Furthermore, all the metal complexes exhibited excellent in vitro anti-inflammatory activity. The Zn(II) complex, in particular, demonstrated significant anti-inflammatory properties surpassing those of the other synthetic metal complexes, with the following order of activity: [ZnL(NO_3_)(H_2_O)_3_] > [CrL(NO_3_)_2_(H_2_O)_2_] > [CuLNO_3_(H_2_O)_3_] > [VOL(H_2_O)OEt] > [LaL(NO_3_)_2_(H_2_O)_2_] > HL. Furthermore, it has been shown that the synthesized complexes effectively bind CT-DNA in an intercalative way, and the binding constant values are in the range of (5.40–6.70) × 10^5^ M^−1^. This indicates strong DNA binding and is equivalent to the value of ethidium bromide, which is 10^7^ M^−1^. The binding free energy of the interaction compounds with the receptor of *Candida albicans* (PDB ID: PDB ID: 5V5Z) agree well with the experimental results.

We investigated the photocatalytic effect of the [CuLNO_3_(H_2_O)_3_] complex in the presence of GO (graphene oxide). Our findings demonstrated that under visible irradiation, the prepared complex exhibited the ability to activate hydrogen peroxide for the degradation of methylene blue, achieving a conversion rate of 65% within 50 min.

This study provides valuable insights into the synthesis, characterization, and diverse applications of the Schiff base ligand and its metal complexes. Further investigation can be conducted to explore the potential biological activities and therapeutic applications of the synthesized metal complexes. This includes studying their cytotoxicity, and potential as anticancer agents. Moreover, the photocatalytic activity demonstrated by the [CuLNO_3_(H_2_O)_3_] complex in the presence of graphene oxide opens up possibilities for developing efficient photocatalysts for environmental remediation and wastewater treatment. Overall, these avenues of research hold promise for the development of new therapeutic agents, environmental solutions, and advanced materials.

## Figures and Tables

**Table 1:** The novel Cu(II), Zn(II), VO(II), Cr(III), and La(III) complexes, as well as their new HL ligand, have the following elemental analysis results and physical characteristics.

Compound	M. Wt.	Color	M. P.	Λ_M_ Ω^−1^ cm^2^ mol^−1^	N%	H%	C%
HL	297.74	Orange-red	180	-	4.70 (4.51)	4.06 (4.55)	68.58 (68.3)
VOL	426.68	Dark green	>300	9.62	6.31 (2.97)	2.95 (3.97)	46.0 (47.3)
CrL	508.74	Deep green	>300	12.76	8.26 (9.12)	2.97 (5.59)	40.0 (41.54)
CuL	488.24	Black	>300	15.30	5.70 (5.86)	4.10 (4.34)	44.01 (44.20)
LaL	595.67	Dark brown	>300	11.20	7.05 (7.45)	2.54 (2.76)	34.28 (35.00)
ZnL	472.12	Brown	>300	19.30	5.86 (5.76)	4.09 (4.04)	43.83 (43.50)

**Table 2:** The characteristic IR bands (cm^−1^) of the Schiff base ligand and its new metal compounds.

Compounds	*v*_(OH)/H_2_O_	*v*_(C=N)_	*v*_(C-O)_	*v*_(NO_3_)_	*v*_(H_2_O)Coor._	*v*_(M-O)_	*v*_(M-N)_
HL	3299	1658	1241		*—*	—	*—*
CuL	3444	1597	1256	1372	947	591	442
ZnL	3327	1614	1237	1381	974	573	457
VOL	3384	1611	1272	1364	971	596	459
CrL	3381	1608	1239	1375	943	659	432
LaL	3385	1622	1244	1382	974	570	456

**Table 3:** Electronic spectral data λ_max_ (nm) of the ligand (HL) and its metal complexes.

Imine Ligand and Its Complexes	λ_max_ (nm)	ε (dm^3^ mol^−1^ mm^−1^)	Assignment
HL	287	1890	π→π*
	334	1630	n→π*
ZnL	289	1410	π→π*
	360	1220	n→π*
	484	1440	LMCT band
VOL	289	1490	π→π*
	360	1290	n→π*
	486	1650	LMCT band
	532	1470	d-d band
CrL	288	1680	π→π*
	353	150	n→π*
	430	1730	LMCT band
	535	1770	d-d band
CuL	288	1640	π→π*
	355	1450	n→π*
	488	1910	LMCT band
	550	1890	d-d band
LaL	288	1680	π→π*
	359	1500	n→π*
	430	1740	Intra-ligand band
	490	1250	LMCT band

**Table 4:** The [CuLNO_3_(H_2_O)_3_] and [ZnLNO_3_(H_2_O)_3_] complexes have the following significant optimized bond lengths (Å) and bond angles (°).

Bond Lengths of [CuLNO_3_(H_2_O)_3_]		Bond Lengths of [ZnLNO_3_(H_2_O)_3_]	
Cu-N1 = 2.006	Cu-O3 = 2.096	Zn-N1 = 2.219	Zn-O3 = 2.112
Cu-O1 = 1.931	Cu-O4 = 2.080	Zn-O1 = 2.013	Zn-O4 = 2.102
Cu-O7 = 2.466	Cu-O8 = 2.322	Zn-O7 = 2.247	Zn-O8 = 2.237
**Bond Angles of [CuLNO_3_(H_2_O)_3_]**		**Bond Angles of [ZnL(H_2_O)_3_NO_3_]**	
N1-Cu-O1 = 93.70	O8-Cu-N1 = 104.8	N1-Zn-O1 = 87.48	O8-Zn-N1 = 100.1
N1-Cu-O3 = 95.38	O8-Cu-O1 = 93.71	N1-Zn-O3 = 95.51	O8-Zn-O1 = 92.75
O1-Cu-O4 = 83.15	O8-Cu-O3 = 88.42	O1-Zn-O4 = 83.85	O8-Zn-O3 = 87.00
O3-Cu-O4 = 86.99	O8-Cu-O4 = 81.18	O3-Zn-O4 = 93.14	O8-Zn-O4 = 85.90
O7-Cu-N1 = 92.73	N1-Cu-O4 = 169.8	O7-Zn-N1 = 89.38	N1-Zn-O4 = 169.65
O7-Cu-O1 = 100.3	O1-Cu-O3 = 169.5	O7-Zn-O1 = 98.69	O1-Zn-O2 = 177.0
O7-Cu-O3 = 73.93	O7-Cu-O8 = 165.9	O7-Zn-O3 = 81.10	O7-Zn-O8 = 165.9
O7-Cu-O4 = 78.31	O1-N1-O3-O4 = 3.620 *	O7-Zn-O4 = 86.37	O1-N1-O3-O4 = 3.698 *

* dihedral angle.

**Table 5:** Important optimized bond lengths (Å) and bond angles (°) of [CrL(NO_3_)_2_(H_2_O)_2_] and [LaL(NO_3_)_2_(H_2_O)_2_] complexes.

Bond Lengths of [CrL(NO_3_)_2_(H_2_O)_2_]		Bond Lengths of [LaL(NO_3_)_2_(H_2_O)_2_]	
Cr-N1 = 2.003	Cr-O3 = 2.085	La-N1 = 2.645	La-O3 = 2.636
Cr-O1 = 1.877	Cr-O4 = 2.063	La-O1 = 2.235	La-O4 = 2.474
Cr-O5 = 1.993	Cr-O8 = 2.019	La-O5 = 2.359	La-O6 = 2.427
**Bond Angles of [CrL(NO_3_)_2_(H_2_O)_2_]**		**Bond Angles of [LaL(NO_3_)_2_(H_2_O)_2_]**	
N1-Cr-O1 = 90.58	O6-Cr-N1 = 93.12	N1-La-O1 = 70.09	O6-La-N1 = 102.1
N1-Cr-O3 = 94.01	O6-Cr-O1 = 95.18	N1-La-O3 = 89.62	O6-La-O1 = 98.64
O1-Cr-O4 = 83.15	O6-Cr-O3 = 85.11	O1-La-O4 = 94.69	O6-La-O3 = 102.2
O3-Cr-O4 = 87.63	O6-Cr-O4 = 91.25	O3-La-O4 = 96.09	O6-La-O4 = 76.89
O5-Cr-N1 = 86.13	N1-Cr-O4 = 175.4	O5-La-N1 = 99.8	N1-La-O4 = 171.2
O5-Cr-O1 = 92.68	O1-Cr-O3 = 175.4	O5-La-O1 = 88.68	O1-La-O3 = 153.6
O5-Cr-O3 = 87.09	O5-Cr-O9 = 172.1	O5-La-O3 = 81.15	O5-La-O6 = 151.7
O5-Cr-O4 = 89.70	O1-N1-O3-O4 = 2.692 *	O5-La-O4 = 84.39	O1-N1-O3-O4 = −7.102 *

* dihedral angle.

**Table 6:** Bond angles (°) and lengths (Å) of the [VOL(H_2_O)OEt] complex that have been adjusted are significant.

Bond Lengths of [VOL(H_2_O)OEt]		Bond Angles of [VOL(H_2_O)OEt]	
V-N = 2.096	V-O4 = 2.148	N-V-O1 = 88.39	O5-V-N = 98.80
V-O1 = 1.946	V = O5 = 1.619	N-V-O3 = 90.69	O5-V-O1 = 117.4
V-O3 = 1.821		O1-V-O4 = 75.90	O5-V-O3 = 119.9
		O3-V-O4 = 91.70	O5-V-O4 = 94.78

**Table 7:** Calculated energies and properties of HL, [CuLNO_3_(H_2_O)_3_], [ZnLNO_3_(H_2_O)_3_], [CrL(NO_3_)_2_(H_2_O)_2_], [LaL(NO_3_)_2_(H_2_O)_2_], and [VOL(H_2_O)OEt].

Property	HL	CuL	ZnL	CrL	LaL	VOL
E (a.u.)	−1320.698	−2025.642	−1895.130	−2120.412	−2469.228	−1697.446
HOMO (eV)	−5.8812	−6.0412	−5.7705	−6.4853	−6.4886	−5.6687
LUMO (eV)	−2.1415	−3.4599	−2.0836	−3.4090	−2.8474	−2.3756
E_g_ (eV)	3.7397	2.5813	3.6869	3.0763	3.6412	3.2931
Dipole moment (Debye)	4.8667	4.0548	3.2068	9.8112	9.0395	3.0716
I = −E_HOMO_	5.8812	6.0412	5.7705	6.4853	6.4886	5.6687
A = −E_LUMO_	2.1415	3.4599	2.0836	3.409	2.8474	2.3756
χ = (I + A)/2	4.0114	4.7506	3.9271	4.9472	4.668	4.0222
η = (I − A)/2	1.8699	1.29065	1.8435	1.5382	1.8206	1.6466
S = 1/2η	0.2674	0.3875	0.2712	0.3251	0.2746	0.3037
μ = −χ	−4.0113	−4.7506	−3.9271	−4.9472	−4.668	−4.0222
ω = μ2/2η	4.3027	8.7428	4.1828	7.9558	5.9845	4.9126

**Table 8:** Spectral parameters for the interaction of CT-DNA with the new complexes.

Complex	λ _max_ Free (nm)	λ _max_ Bound (nm)	∆n	Chromism (%) ^a^	Type of Chromism	K_b_ × 10^5^ (mol^−1^dm^−3^)	∆G (k J mol^−1^)
HL	287	290	−2	22	Hypo		
	334	336	−2	21			
	359	354	5	24		2.2	−30.5
	429	443	−4	17			
	493	482	9	−3.3			
ZnL	289	291	−2	22			
	360	362	−2	26			
	396	399	−3	31	Hypo	7.3	−33.5
	431	434	−3	24			
	484	489	−5	19			
	523	529	−6	13			
VOL	289	292	−3	8			
	334	336	−2	9			
	360	352	8	10	Hypo		−32.9
	398	400	−2	15		6	
	430	441	−11	6			
	486	484	2	18			
	525	523	2	23			
CrL	288	290	−2	6			
	333	335	−2	7			
	353	350	3	7	Hypo	4.4	−32.2
	397	399	−2	6			
	484	488	−4	31			
CuL	288	289	−1	30			
	355	351	4	35	Hypo	5.4	−32.7
	430	432	−2	31			
	488	485	3	40			
	532	524	8	49			
LaL	288	290	−1	15			
	334	332	2	17	Hypo	6.7	−33.3
	359	361	−2	16			
	398	399	−2	14			
	430	432	−2	11			
	490	486	4	−43			
	535	523	12	−63			

Chromism % a=Absfree−AbsboundAbsfree.

**Table 9:** The docking interaction data calculations of HL, [CuL(NO_3_)(H_2_O)_3_], [ZnL(NO_3_)(H_2_O)_3_], [CrL(NO_3_)_2_(H_2_O)_2_], [LaL(NO_3_)_2_(H_2_O)_2_], and [VOL(H_2_O)OEt] with the active sites of the receptor of *Candida albicans* oxidoreductase/oxidoreductase inhibitor (PDB ID: PDB ID: 5V5Z).

	Receptor	Interaction	Distance (Å) *	E (kcal/mol)
**HL**				
O 18	O MET 508	H-donor	2.81 (2.09)	−1.5
**[CuLNO_3_(H_2_O)_3_]**				
O 35	O ARG 44	H-donor	3.70 (2.73)	−0.6
O 41	O SER 42	H-donor	2.67 (1.87)	−17.5
6−ring	CA LEU 43	pi-H	4.08	−0.6
**[ZnL(NO_3_)(H_2_O)_3_]**				
O 35	OD1 ASP 225	H-donor	2.60 (1.59)	−26.8
O 44	OD1 ASP 225	H-donor	2.77 (1.82)	−7.8
O 44	OD2 ASP 225	H-donor	2.79 (1.97)	−2.8
C l22	O HIS 310	H-donor	3.14	−0.6
O 35	OD1 ASP 225	Ionic	2.60	−7.8
O 44	OD1 ASP 225	Ionic	2.77	−6.2
O 44	OD2 ASP 225	Ionic	2.79	−6.0
**[CrL(NO_3_)_2_(H_2_O)_2_]**				
O 34	OD2 ASP 173	H-donor	2.85 (1.96)	−23.4
O 35	OE2 GLU 174	H-donor	2.64 (1.98)	−4.4
Cl 22	NZ LYS 177	H-acceptor	3.40	−0.8
O 34	OD2 ASP 173	Ionic	2.85	−5.5
O 34	OE1 GLU 194	Ionic	2.91	−5.1
O 35	OD2 ASP 173	Ionic	2.90	−5.2
O 35	OE1 GLU 174	Ionic	3.70	−1.2
O 35	OE2 GLU 174	Ionic	2.64	−7.4
**[LaL(NO_3_)_2_(H_2_O)_2_]**				
O 21	O SER 442	H-donor	3.00 (2.02)	−3.0
O 34	OE1 GLU 444	H-donor	3.27 (2.39)	−2.0
**[VOL(H_2_O)OEt]**				
O 34	NZ LYS 382	H-acceptor	3.01 (2.02)	−21.5

* The lengths of H-bonds are in brackets.

## Data Availability

Data are contained within the article.

## Supplementary Materials

The following supporting information can be downloaded at: https://www.mdpi.com/article/10.3390/molecules28124777/s1, Figure S1: FT-IR spectra of HL ligand and its new CuL, ZnL, VOL, CrL and LaL complexes, Figure S2: EI-mass spectral analysis of HL ligand and its CrL complex, Figure S3: UV-vis. Absorption spectra of 10^−3^ M solution of the HL ligand and its VOL, CuL, CrL, ZnL and LaL in DMF, Figure S4: Powder XRD data of CrL, ZnL and VOL complexes, Figure S5: Powder XRD data of CuL complex, Figure S6: TGA study of the HL ligand and its CuL, ZnL, VOL, CrL, and LaL complexes at temperatures between 25 and 1000 °C using nitrogen and a heating rate of 5 °C per minute and Figure S7: Inhibition zone in mm for the new complexes. Table S1: For the novel complexes, the following information is provided: thermal degradation phases, mass losses, projected lost segments, final residue, and thermos-kinetic activation parameters of each breakdown step in the temperature range of 25 to 1000 °C under N2, a heating rate of 5 °C/min, Table S2: Minimum inhibition concentration (MIC) μg/ml for antibacterial assay of the new Schiff base ligand and its complexes and section Thermogravimetric analysis.
