# Supplementary material for: Synthesis, Characterization, DFT Studies of Novel Cu(II), Zn(II), VO(II), Cr(III), and La(III) Chloro-Substituted Schiff Base Complexes: Aspects of Its Antimicrobial, Antioxidant, Anti-Inflammatory, and Photodegradation of Methylene Blue
Source: Molecules. 2023 Jun 15;28(12):4777. doi: 10.3390/molecules28124777 (PMC10301192; doi:10.3390/molecules28124777)
Supplement: Supplementary file 1 [file molecules-28-04777-s001.zip › molecules-2396622-supplementary.pdf]

Supplementary Materials

Synthesis, Characterization, DFT Studies of Novel Cu(II), Zn(II), VO(II), Cr(III), and La(III) Chloro-Substituted Schiff Base Complexes: Aspects of Its Antimicrobial, Antioxidant, Anti-Inflammatory, and Photodegradation of Methylene Blue

Laila H. Abdel-Rahman [1,*], Maram T. Basha [2], Badriah Saad Al-Farhan [3,*], Walaa Alharbi [4], Mohamed R. Shehata [5], Noura O. Al Zamil [6] and Doaa Abou El-ezz [7]

[1] Chemistry Department, Faculty of Science, Sohag University, Sohag 82534, Egypt
[2] Department of Chemistry, College of Science, University of Jeddah, Jeddah 21589, Saudi Arabia; mtbasha@uj.edu.sa
[3] Chemistry Department, Faculty of Girls for Science, King Khalid University, Abha 61421, Saudi Arabia
[4] Department of Chemistry, Science and Arts College, King Abdulaziz University, Rabigh 21911, Saudi Arabia; wnhalharbe@kau.edu.sa
[5] Chemistry Department, Faculty of Science, Cairo University, Giza 12613, Egypt; mrshehata@sci.cu.edu.eg
[6] Department of Chemistry, College of Science, Imam Abdurrahman Bin Faisal University, P.O. Box 1982, Dammam 31441, Saudi Arabia; nalzamil@iau.edu.sa
[7] Department of Pharmacology and Toxicology, Faculty of Pharmacy, October University for Modern Sciences and Arts (MSA University), Giza 12566, Egypt; dabulez@msa.edu.eg
* Correspondence: laila.abdelrahman@science.sohag.edu.eg (L.H.A.-R.); shahd_bb@hotmail.com (B.S.A.-F.)

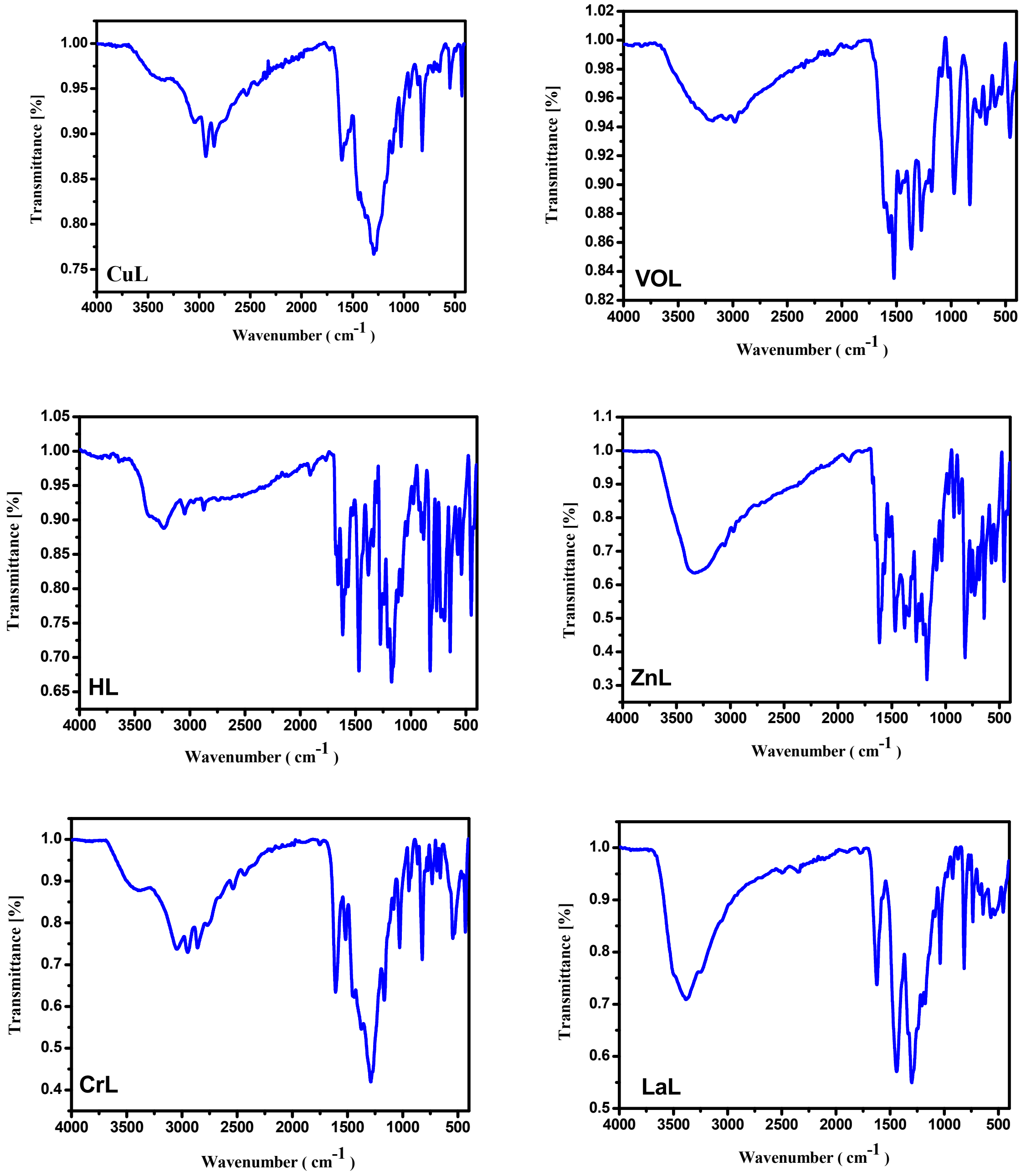

Figure S1: FT-IR spectra of HL ligand and its new CuL, ZnL, VOL, CrL and LaL complexes.

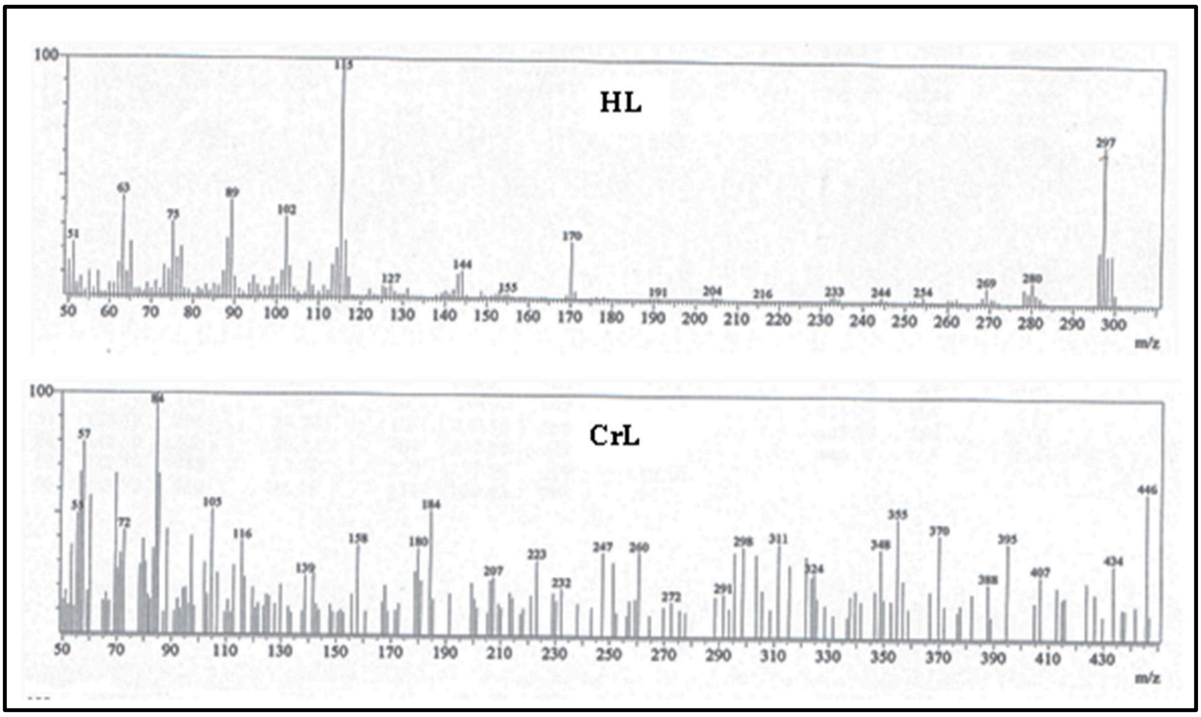

Figure S2: EI-mass spectral analysis of HL ligand and its CrL complex.

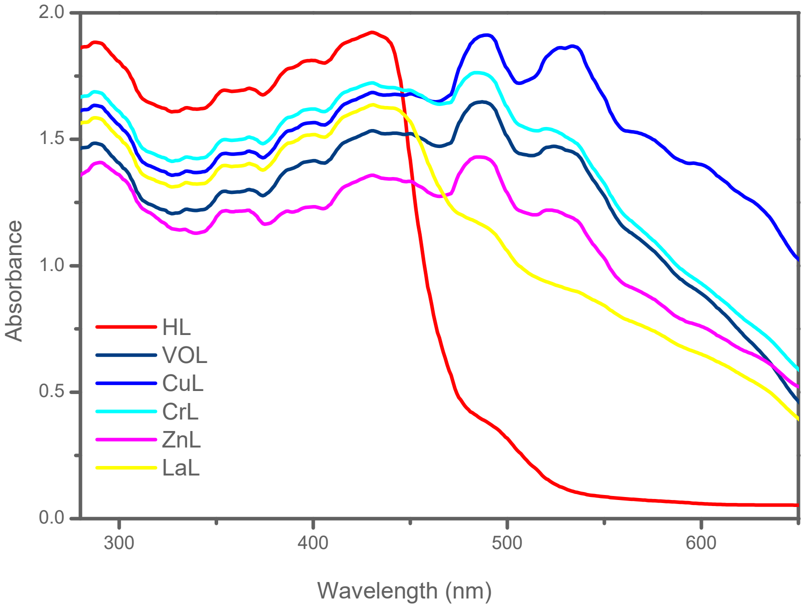

Figure S3: UV-vis. Absorption spectra of 10^{-3} M solution of the HL ligand and its VOL, CuL, CrL, ZnL and LaL in DMF.

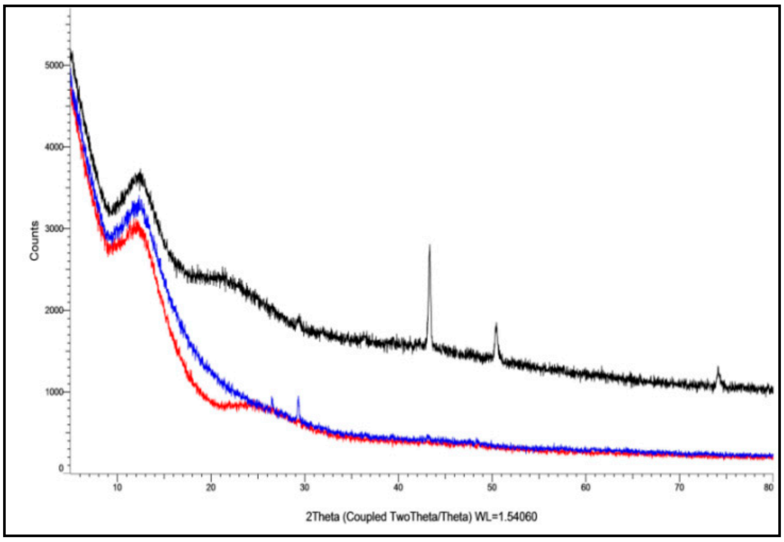

Figure S4: Powder XRD data of CrL, ZnL and VOL complexes.

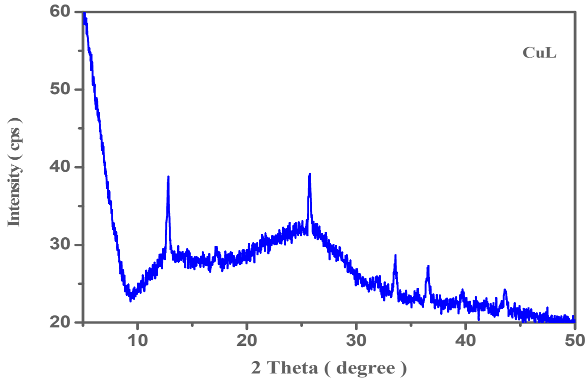

Figure S5: Powder XRD data of CuL complex.

Thermogravimetric analysis

The thermal stability of the new ligand HL and its CuL, ZnL, VOL, CrL, and LaL complexes was inspected by utilizing TG and DTG techniques within the temperature range of 25-1000 °C[13], [29]. The obtained results are shown in **Figure S6** and listed in **Table S1**. The Schiff base ligand HL showed two decomposition steps in the temperature range 190.24 - 303.16 °C and 303.16 - 883.33 °C, the first step was accompanied by weight loss of 19.67 % and the second step by 78.60 % weight loss. The first loss could be due to the loss of two OH groups and one HCl from the Schiff base ligand. The second loss corresponds to the loss of the remaining part from the Schiff base ligand $C_{18}H_{17}N$ calculated loss is 79.01 % (exp. 78.60 %). Five stages of breakdown were visible in the ZnL complex. The first step starts when the temperature is between 27 and 153 °C, which is consistent with the loss of three H_2O, one HNO_3, and HCl (Found at 31.05 and Calculated at 30.92%). At 118.07°C, the

temperature reached its highest value. The second stage occurred between 153-246 °C, which corresponded to the loss of the fragment C_3H_6O (Found = 10.69% and calculated = 11.76%). The peak temperature was 146.4 °C. The third stage, which occurred between 246-330 °C (Found = 8.16% and calculated = 7.63%), reflected the loss of the C_3H_6 molecule. The highest temperature reached a peak of 226 °C. The loss of the C_4H_8 molecule in the fourth stage (Found = 10.32% and Calc. = 11.37%) occurred in the temperature range of 330 – 432 °C. The highest temperature reached a peak of 226 °C. The loss of the C_4H_8 molecule in the fourth stage (Found = 10.32% and Calc. = 11.37%) occurred in the temperature range of 330 – 432 °C. 314 °C was the highest temperature for the day. At 432 - 626 °C, the fifth phase occurred, and the loss of the C_7H_4N molecule (Found = 20.65% and calculated = 21.52%) followed. The highest temperature reached a height of 513 °C. Finally, the weight reduction totaled 80.40% and the zinc oxide remained available as residue. Three stages of disintegration were visible in the CrL complex TG thermogram. The initial stage starts at a temperature between 54 and 134 °C, which is consistent with the H_2O loss (Found at 3.01 and calculated at 3.40. At 74.15 °C, the highest temperature was reached. The loss of $2H_2O$ and $2HNO_3$ was represented by the second phase, which took place in the 134–300 °C temperature range (Found = 31.11% and calculated = 30.40%). The highest temperature recorded was 213.2 °C. The loss of the $C_{17}H_{12}ClNO$ molecule in the third stage (Found = 52.60% and Calc. = 53.20%; temperature range: 300–1000 °C) was represented by this. The maximum temperature peak was at 226 °C. Finally, the weight loss caused by the 0.5 Cr_2O_3 residues came to (Found = 13.23% and Calc. = 14.41%). The VOL complex's TG curve revealed three stages of deterioration. Temperatures between 36 and 206 °C are required for the first step, which is preceded by a weight loss of 6.80 % (Calc. 6.33 %) that may be attributed to the loss of the CH_2O moiety from the Schiff base ligand. In the temperature range of 206-350°C, a weight of 19.54 % (calc. 19.09 percent) has been removed from the VOL complex. The elimination of the C_5H_9N moiety from the ligand may be responsible for this loss. The third stage occurred between 350 and 833 °C and was accompanied by a weight loss of 52.52 % (52.07 % in calculations), which may have been caused by the elimination of the HCl and $C_{13}H_6O_2$ moieties from the ligand, leaving a VO_2 residue. Three stages of decomposition were visible in the CuL complex's TG thermogram. The first step starts at a temperature between 29 and 174 °C, which is consistent with the loss of one hydrated water molecule (H_2O) (Found 3.38 and calc. 3.64 %). The second stage took place in the 134–365 °C temperature range and resulted in the loss of three H_2O, one HCl, and one HNO_3 (Found = 31.89 % and calculated = 30.22 %). The highest recorded temperature was 201.83 °C. In the third step, the loss of the $C_{17}H_{13}NO$ molecule (found to be 49.20 % and calculated to be 48.64 %) was indicated by temperatures between 367 and 799 °C. Finally, the CuO was still present as residues with weight loss of (Found = 17.29 % and calc. = 16.65 %).

LaL thermogram revealed five stages of degradation. When the temperature is between 29 and 174 °C, the first step starts, which is consistent with the loss of two coordinated H_2O molecules and one hydrated H_2O molecule (Found at 9.06 and calculated at 9.08%). The loss of the fragment HNO_3 (Found = 10.58% and calculated = 10.59%) occurred in the second phase, which took place in the temperature range of 174 to 229 °C. The loss of an HCl molecule in the third stage (Found = 6.13% and calculated = 6.13%) occurred in the temperature range of 229–267 °C. The loss of the naphthaldehyde fragment $C_{10}H_7O$ in the fourth stage (Found = 24.01% and calculated = 24.03%) occurred in the temperature range of 267 to 630 °C. Loss of HNO_3 and CH_2N fragments (Found = 15.28% and calc. = 15.30) occurred in the fifth stage, which occurred between 630 and 1000 °C. The remaining residues of lanthanum oxide and C_6H_6 totaled 39.12% (calculated as 39.15%) of the original weight. The thermodynamic activation parameters of decomposition processes have been determined and presented in **Table 4** using the Coats-Redfern equation. The energy of activation of the synthesized compounds is positive as a result, indicating that the degradation processes are endothermic. The activation entropy of the formed complexes is negative, indicating that they are more actively ordered than the reactant and that the process is delayed. Bond polarization and electronic transitions in the activated state may be the cause of the reaction's slow start. The values of the free energy change (ΔG^*) in metal complex degradation reactions are high positive, suggesting that the breakdown stages are not spontaneous processes in which the free energy of the starting molecule is lower than that of the end product [26], [30], [31].

Table S1: For the novel complexes, the following information is provided: thermal degradation phases, mass losses, projected lost segments, final residue, and thermos-kinetic activation parameters of each breakdown step in the temperature range of 25 to 1000 °C under N_2, a heating rate of 5 °C/min.

Complexes	Temperature °C	Fragment loss %		Weight loss %		E^* (kJmol^{-1})	A (S^{-1})	ΔH^* (kJmol^{-1})	ΔG^* (kJmol^{-1})	ΔS^* (Jmol^{-1}K^{-1})
		Lost form	M. Wt.	Found	Calc.					
ZnL	27 – 153 °C	$3H_2O + HNO_3 + HCl$	153.5	31.049	30.92			- 2438	13984	-50.1
	153 – 246 °C	C_3H_6O	58.0	10.687	11.76			- 3232	14098	-52.4
	246 – 330 °C	C_3H_6	42.0	8.16	7.63	6.5	8.9	-3598	14234	-54.3
	330 – 432 °C	C_4H_8	56.0	10.319	11.37			-4984	14987	-54.2
	432 – 626 °C	C_7H_4N	102.0	20.651	21.52			-5430	15987	-55.1
Residue	>800 °C	ZnO	81.38	19.693	16.50					
CrL	54 – 134 °C	H_2O hyd.	18.0	3.014	3.4			-1098	22876	-51.1
	134 – 300 °C	$2H_2O$coord. + $2HNO_3$	160.0	31.11	30.4	2.2	3.2	-1703	22989	-52.3
	300 -1000 °C	$C_{17}H_{12}ClNO$	280.5	52.60	53.2			-2349	23098	-53.2
Residue	>800 °C	0.5 Cr_2O_3	75.9	13.267	14.41					
VOL	36 – 206 °C	CH_2O	27.0	6.796	6.33			-1293	12872	-45.2
	206 – 350 °C	C_5H_9N	81.5	19.454	19.09	6.1	7.8	-1394	12987	-47.5
	350 -833 °C	$HCl + C_{13}H_6O_2$	36.5+ 194.0	52.522	52.07			-1598	13987	-48.1
Residue	>800 °C	VO_2	82.9	21.228	19.43					
CuL	39 – 134 °C	H_2O hyd.	18.00	3.382	3.64			-4056	24098	-51.4
	134 – 365 °C	$3H_2O + HNO_3$ - HCl	153.50	30.886	30.22	7.4	6.9	-4505	24145	-52.6
	365 – 799 °C	$C_{17}H_{13}NO$	247.00	49.20	48.64			-5096	24678	-54.3
Residue	>750 °C	CuO	31.75	17.29	15.65					
	29 – 174 °C	$2H_2O$coord. + H_2O hyd.	54	9.06	9.08			-1983	23098	-56.7
LaL	174 – 229 °C	HNO_3	63	10.58	10.59			-2045	23765	-57.8
	229 – 267 °C	HCl	36.5	6.13	6.13	11.7	15.9	-2345	23987	-58.1
	267 – 630 °C	$C_{10}H_7O$	143	24.01	24.04			-2798	24987	-58.3
Residue	630 – 1000 °C	$HNO_3 + CH_2N$	91	15.28	15.30			-2875	25310	-58.5
	>1000°C	LaO + C_6H_6	219	39.12	39.15					

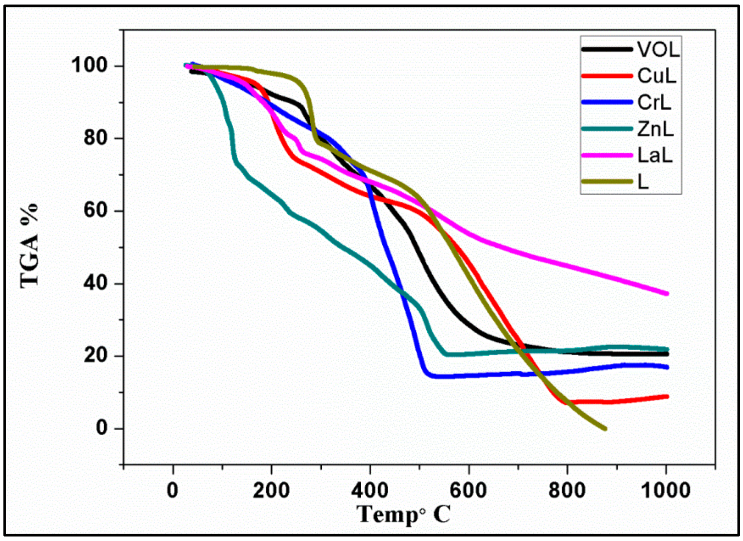

Figure S6: TGA study of the HL ligand and its CuL, ZnL, VOL, CrL, and LaL complexes at temperatures between 25 and 1000 °C using nitrogen and a heating rate of 5 °C per minute.

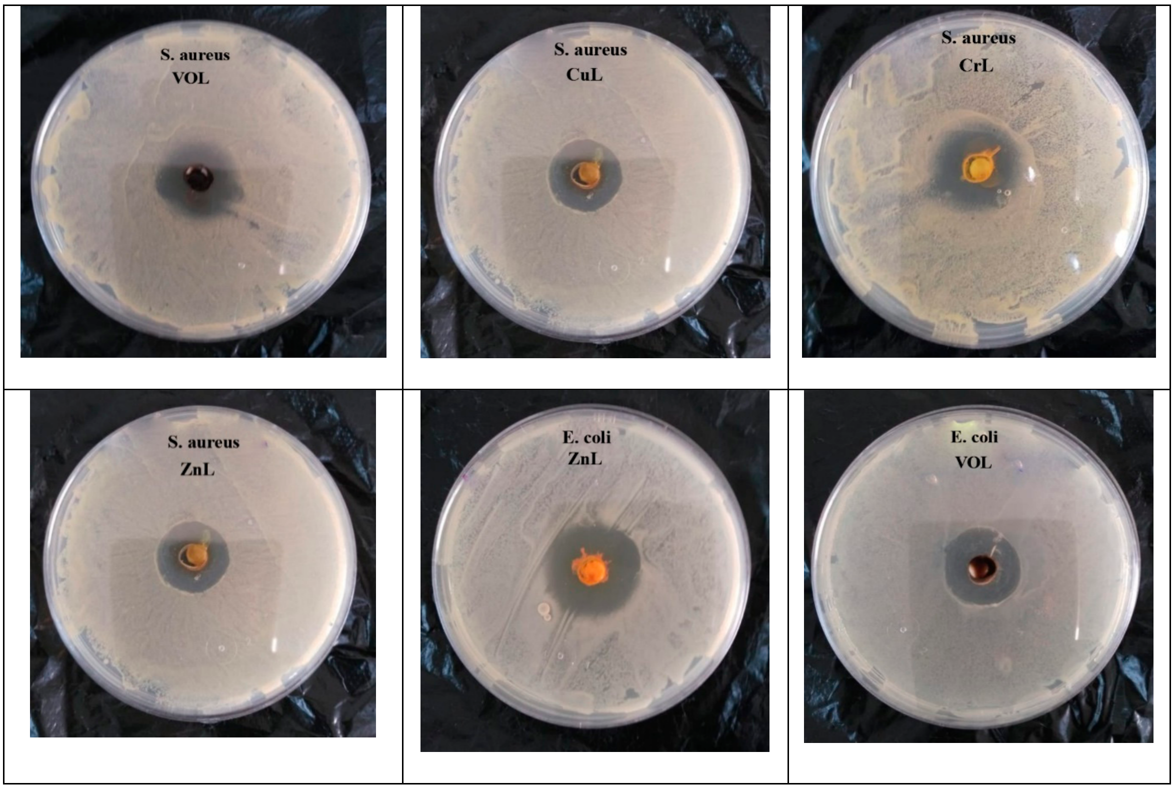

Figure S7: Inhibition zone in mm for the new complexes.

Table S2: Minimum inhibition concentration (MIC) µg/ml for antibacterial assay of the new Schiff base ligand and its complexes

	HL	LaL	ZnL	VOL	CuL	CrL	Ampicillin	Nystatin	Gentamicin
S. *aureus*	26.0	19.6	21.5	19.3	16.6	12.3	20.0		
E. *coli*	28.5	19.5	25.3	19.0	15.3	14.5			27.0
C. *albicans*	16.5	39.3	49.6	30.6	12.7	9.6		21.0	
A. *niger*	0	0	18.6	0	0	0		8.0	
